# H19 encourages aerobic glycolysis and cell growth in gastric cancer cells through the axis of microRNA-19a-3p and phosphoglycerate kinase 1

**DOI:** 10.1038/s41598-023-43744-0

**Published:** 2023-10-11

**Authors:** Siche Chen, Haiping Wang, Peiren Xu, Shengchun Dang, Yongqin Tang

**Affiliations:** 1 Department of Gastrointestinal Surgery, Affiliated Hospital of Jiangsu University, Zhenjiang, 212000 China; 2 Department of Emergency Surgery, the Second Hospital of Anhui Medical University, Hefei, 230001 Anhui China; 3 School of Stomatology, Zhengzhou University, Zhengzhou, 450001 Henan China; 4 Siyang Hospital, Suqian, 223700 Jiangsu China; 5 Department of Hepatobiliary Surgery, Affiliated Drum Tower Hospital, Medical School of Nanjing University, Nanjing, 210008 China

**Keywords:** Cancer, Cell biology

## Abstract

Numerous studies have been conducted on long non-coding RNAs (lncRNAs) in human tumors like gastric cancer (GC). Our research uncovers how aerobic glycolysis and cell proliferation in gastric cancer cells are related to H19. We discovered that H19 was highly expressed in tumor tissues and that patients with higher H19 expression have a poorer prognosis. Intriguingly, we applied the subcellular isolation, luciferase reporter, western blot analysis, MTT, colony formation experiments, and CDX Model in Mice to verify that H19 regulates aerobic glycolysis towards GC cell growth by H19/microRNA (miR)-19a-3p/phosphoglycerate kinase 1 (PGK1) axis. Together, our research offers proof that the H19/miR-19a-3p/PGK1 pathway aids in the regulation of aerobic glycolysis and cell proliferation in GC. This may offer an opportunity for novel therapeutic approaches to the treatment of GC.

## Introduction

With an estimated 768,793 deaths reported in 2020, gastric cancer is the fifth most frequently diagnosed cancer globally and accounts for 6% of all new cancer cases each year. In comparison to other parts of the globe, east Asia has a significantly higher incidence of gastric cancer. (https://gco.iarc.fr/today/data/factsheets/cancers/7-Stomach-fact-sheet.pdf).

RNA with a length greater than 200 nucleotides but no role in protein-coding is known as long noncoding RNA^1^. Recent research has demonstrated that lncRNAs are involved in a variety of human cancers and their biological processes, including development, metastasis, drug tolerance, metabolism, and immune escape^2–5^. H19 is a gene for a long noncoding RNA, that has been reported to play a significant role in various human tumors, including breast cancer, colorectal cancer, pancreatic cancer, and GC^6–10^. Zhang et al. observed that H19 was overexpressed in GC tissues compared to nearby normal tissues and its overexpression level was positively correlated with overall survival time^11^. Additionally, research has shown that H19 significantly controls GC cell proliferation, migration, and invasion^12,13^. However, the molecular mechanism of H19 regulating gastric cancer has not been elucidated in detail.

The Warburg effect, also known as aerobic glycolysis, is a hallmark of cancer that gives cancer cells an advantage over healthy cells in terms of development by supplying energy and building blocks for biosynthesis even in the presence of plenty of oxygen^14^. Although aerobic glycolysis is now generally acknowledged and used as a therapeutic target for cancer, its molecular underpinnings remain obscure^15^. Increasing data suggest that lncRNAs can modulate signaling pathways or directly control the glycolytic enzymes to control glucose metabolism in cancer cells^16^. For instance, by maintaining PFKFB3, lncRNA AGPG increased glycolysis activity and cell proliferation in esophageal squamous cell carcinoma^17^. lncRNA MACC1AS1 enhanced GC cell metabolic plasticity through AMPK/Lin28-mediated mRNA stability of MACC1^18^. Phosphoglycerate kinase 1 (PGK1) is an important enzyme in glycolysis and generates the first ATP in the glycolysis pathway. It has been reported that lncRNA, called LINC01559, promoted the proliferation of gastric cancer via upregulating PGK1^19^. However, the relationship between H19 and pgk1 in gastric cancer has not yet been reported.

In this investigation, we found that the levels of H19 were markedly elevated in GC tissue specimens and were related to a bad prognosis. Mechanistically, H19 regulates the glycolysis of gastric cancer cells to affect cell proliferation through H19/MiR-19a3p /PGK1aix in gastric cancer.

## Materials and methods

### Human tissue samples

Between January 2017 and December 2020, 50 pairs of fresh tumor tissue specimens and nearby normal tissue specimens were collected from GC patients at the Affiliated Hospital of Jiangsu University (Zhenjiang, China). All tissue samples were quickly frozen in liquid nitrogen. The tissue samples taken from patients were approved by the ethical review committee of the Affiliated Hospital of Jiangsu University, Zhenjiang, China. We confirm that all methods were performed in accordance with the relevant guidelines and regulations. Patients who underwent radiotherapy and chemotherapy before surgery were excluded from this study. All patients were informed of the risks and benefits of the experimental study, and the informed consent of all patients has been signed.

### Cell culture

MKN-45, SGC-7901, and HEK293T cell lines were obtained from American Type Culture Collection (ATCC). HEK293T cells were cultured in DMEM with 1% Pen/Strep solution and 10% FBS in 5% CO2 at 37 °C. MKN-45 and SGC-7901 cells were grown in DMEM with 1% Pen/Strep solution and 10% FBS in 5% CO2 at 37 °C.

### RNA isolation and RT‐qPCR

Utilizing the TRIZOL reagent from Invitrogen, total cellular RNA was extracted and then converted into complementary DNA (cDNA). For RT-qPCR, SYBR Green PCR Master Mix (Takara, Kyoto, Japan) was used. The 2CT method was used to calculate gene expression, normalizing to Tubulin. The qPCR primers used in this research can be found in Table 1.

**Table 1:** Relative primers used for PCR, real-time PCR, stem-loop sequences (All primer sequences are from human).

Name	Sequences
U6	F: CTCGCTTCGGCAGCACA R: AACGCTTCACGAATTTGCGT
H19	F: GGAGTGAATGAGCTCTCAGG R: CTAAGGTGTTCAGGAAGGCC
GAPDH (Glyceraldehyde 3-phosphate dehydrogenase)	F: ACAACTTTGGTATCGTGGAAGG R: GCCATCACGCCACAGTTTC
β-Tubulin	F: CGGGCAGTGTTTGTAGACTTGG R: CTCCTTGCCAATGGTGTAGTGC
PGK1 (Phosphoglycerate kinase 1)	F: TGGACGTTAAAGGGAAGCGG R: GCTCATAAGGACTACCGACTTGG
PDK1 (Pyruvate dehydrogenase lipoamide kinase isozyme 1)	F: CTGTGATACGGATCAGAAACCG R: TCCACCAAACAATAAAGAGTGCT
PFKFB3 (6-phosphofructo-2-kinase/fructose-2,6-biphosphatase 3)	F: TTGGCGTCCCCACAAAAGT R: AGTTGTAGGAGCTGTACTGCTT
SLC2A1 (Solute carrier family 2, facilitated glucose transporter member 1)	F: GGCCAAGAGTGTGCTAAAGAA R: ACAGCGTTGATGCCAGACAG
miRNA-19a-3p	RT: GTCGTATCCAGTGCAGGGTCCGAG GTATTCGCACTGGATACGACACGTGT F: AGTCAAAACGTATCTAA R: GTGCAGGGTCCGAGGT
miRNA-193a-3p	RT: GTCGTATCCAGTGCAGGGTCCGAG GTATTCGCACTGGATACGACGGTCAA F: TGACCCTGAAACATCC R: GTGCAGGGTCCGAGGT
miRNA-19b-3p	RT: GTCGTATCCAGTGCAGGGTCCGAG GTATTCGCACTGGATACGACACGTGT F: AGTCAAAACGTACCTAA R: GTGCAGGGTCCGAGGT

### Western-blot

A normal medium was used to cultivate the cells. When cells achieved 70%–80% confluence, they were washed and lysed in radioimmunoprecipitation assay (RIPA) buffer supplemented with proteinase inhibitor and phosphorylase inhibitor. Scratching on ice was used to collect the lysate. The amount of protein was measured using a Pierce BCA protein assay reagent (Cat. No. 23227, Thermo Fisher). The same quantity of proteins was cooked at 95 °C for 10 min after being combined with 5X Leammli sample solution. The SDS-PAGE gel was used to load the protein, which was then electrophoretically separated and moved to PVDF membranes. Membranes were first stained for 1 min with Ponceau and then blocked for 1 h at room temperature with a blocking solution (PBS + 0.1% Tween + 5% BSA). The membranes were then exposed to the primary antibody for an extended incubation at 4 °C. Table 2 provides information on the main antibodies used in this study. The proper secondary antibody was then applied to the membranes, and they were left to sit at room temperature for an hour. Chemiluminescence was used to detect immunoreactive bands. There were three separate tests conducted.

**Table 2:** List and characteristics of the staffs used for immunohistochemistry, western blot and immunofluorescence.

Antibody	Host	Dilution	Type	Company
PGK1	Rabbit	1:1000	WB	Abcam
β-Tubulin	Rabbit	1:2000	WB	Abcam
Ki67	Rabbit	1:250	IF	Abcam
Ki67	Rabbit	1:1000	IHC	Abcam
HPR-Anti-Mouse	Goat	1:5000	WB	Jackson ImmunoR
HPR-Anti- Rabbit	Goat	1:5000	WB	Jackson ImmunoR
DAPI	–	1:10,000	IF	CST
DAB Peroxidase (HRP) Substrate Kit	–	–	IHC	Abcam
Rabbit specific HRP/DAB (ABC) Detection IHC Kit	–	–	IHC	Abcam

### Colony formation assay

The 6-well dishes were seeded with 3*103 cells per well with the cell samples. After 5 days of culture, samples were successively treated with 4% paraformaldehyde and crystal violet solution. Colonies with more than 50 cells were then manually counted.

### Transfection

Two small interfering RNAs (siRNA) targeting human H19 (si-H19-1, and si-H19-2), were obtained from Thermo Fisher Scientific (Catalog #1,299,001). Negative control (NC), miR-19a-3p mimic, and miR-19a-3p inhibitor were purchased from Thermo Fisher Scientific (Catalog # 4,464,066, Catalog # 4,464,084). According to the manufacturer's recommendations, Lipofectamine 2000 (Invitrogen, Carlsbad, CA, USA) was used to transfect siRNA reagent or plasmids into SGC-7901 and MKN-45 cells.

The p-Lenti-EF1a-Backbone plasmid was used to clone a human H19 RNA transcript, creating a stable H19 overexpression (OE) cell line. BamHI and XbaI are the restriction enzyme binding sites. We selected two distinct shRNAs (built into the PLKO plasmid bought from Thermo Fisher) to create a stable H19 knockdown (KD) cell line. Human PGK1 cDNA clone into a p-Lenti-EF1a-Backbone plasmid and created PGK1-OE plasmid. Table 1 contains a summary of the primers. The NC mimics and miR-19a-3p mimics were both made by Genepharma. Using Lipofectamine 2000, cell transfection with the specified plasmids was carried out for 48 h (Invitrogen). In 6-well dishes, 0.5*106 HEK293T cells were seeded and using Fu-GENE transfection reagent, 2 ug of control, H19-overexpression, and shRNA plasmids along with the packaging plasmid were transfected. The virus supernatant was taken out and transfected into the MKN-45 and SGC-7901 cell lines. 72 h after transfection, cells were selected with 10 ug/ml puromycin for two passages forty-eight hours after transduction and then grown in a regular medium containing 2 ug/ml puromycin.

### Subcellular fractionation

To perform the subcellular fractionation test in GC cell samples, the PARISTM Kit (Ambion, Austin, TX) was purchased and used following the user manual. By using RT-qPCR, the expression levels of H19, PGK1, U6, and GAPDH were examined. The qPCR primers used in this research can be found in Table 1.

### Dual-luciferase reporter assay

To create H19-WT/Mut and PGK1-WT/Mut reporter vectors, fragments of full-length H19 or PGK1 3'untranslated region (3′UTR) having wild-type (WT) or mutated (Mut) miR-19a-3p binding sites were inserted into the pmirGLO Dual-Luciferase Reporter Vector (Promega, Madison, WI). Then, for 48 h, miR-19a-3p mimics or NC mimics were co-transfected with each of the aforementioned four types of hybrid plasmids. Finally, the Dual-Luciferase Reporter Assay System was used to measure the luciferase activities (Promega).

### In vivo tumor growth experiment

Twelve male BALB/c nude mice (Shi Laike Company, Shanghai, China) were randomly allocated to three experimental groups. Mice were xenografted with the MKN-45 cells that had been transfected with sh-NC or sh-H19#1 and the pcDNA3.1 vector harboring H19. The tumor volume in the aforementioned three groups was then evaluated every four days after that. Mice were killed 28 days after injection, and tumor weights were noted for analysis. the ethical review committee of the Affiliated Hospital of Jiangsu University gave its approval before the in vivo research could be carried out. Moreover, this study is reported in accordance with ARRIVE guidelines (https://arriveguidelines.org/).

### Immunohistochemistry (IHC)

Slides were heated at 55 °C for 10 min, then merged in xylene for 5 min, followed by successive dewaxing processes (2 × xylene, 100% ethanol, 75% ethanol, 50% ethanol, 5 min each), for immunohistochemistry staining on paraffin sections. The slides were then microwaved for 10 min to boil in 10 mM citrate solution for antigen retrieval before being cooled to room temperature. The VECTASTAIN Universal Quick HRP Kit (PK-7800; Vector Laboratories, Table 2) was used for the following procedures by the manufacturer's instructions. Staining development was carried out using a DAB Peroxidase (HRP) Substrate Kit (SK-4100; Vector Labs, Table 2). The Axio Imager A2 microscope was used to obtain images.

### Cell viability assay

Following the manufacturer's directions, the MTT assay was used to determine the viability of the cells. 3000 cells were seeded in each well of a 96-well plate. Then, the cells were incubated in 20 uL of MTT solution for 1 h at 37 °C after 7 days. At 490 nm, the optical density was measured. Three replicates were made for each condition.

### Glucose uptake, lactate secretion, ATP production assay, and seahorse assay

Following the manufacturer's directions, we measured glucose uptake, lactate production, and ATP production using the Glucose Uptake Colorimetric Assay Kit (Biovision, Cat#: K676-100), Lactate Assay Kit (Cell Biolabs, Cat#: MET-5012), and ATP colorimetric Assay Kit (Biovision, Cat#: K959). Following the manufacturer's directions, the Seahorse XFe 24 Extracellular Flux Analyzer was used to measure the extracellular acidification rate (ECAR) and oxygen consumption rate (OCR). The Seahorse XFe Glycolysis Stress Test Set (Agilent, Cat#: 103,017–100) was used to calculate the ECAR. The OCR was calculated using the Seahorse XF Cell Mito Stress Test Set (Agilent, Cat#: 103,015–100). In a Seahorse XFe 24-cell culture microplate, 3*103 SGC-7901 cells and 3*103 MKN-45 cells were seeded and left to attach for the night. The following day, the XF DMEM assay medium was used instead of the culture medium (Agilent). Following calibration and baseline observations, cells were incubated for 1 h at 37 °C in a CO_2_-free environment before being measured. Sequential additions of glucose (10 mM), oligomycin (1 uM), and the glycolytic inhibitor 2-DG were used to measure the ECAR readings (50 mM). Serial additions of oligomycin (1.5 M), carbonyl cyanide-4 (trifluoromethoxy) phenylhydrazone (FCCP) (1 M), and a mixture of rotenone (0.5 M) and antimycin A (0.5 M) were used to determine the OCR values. Seahorse XFe 24 Wave software was used to examine the data. These observations were carried out using three independent assays and four repeat wells in each cell line.

### Statistical analyses

The mean value and standard deviation (SD) of the data from separate bio-triplications were used to represent all results. Using Prism version 8.0, the data analysis for each cohort was conducted using the Student's T-test and either a one-way or two-way analysis of variance (ANOVA) (GraphPad Software, La Jolla, CA). The two-sided p-value cutoff for statistical significance was fixed at 0.05.

## Result

### H19 was overexpressed in GC tissue specimens and may regulate glycolytic molecules expression by affecting microRNA (miR)-19a-3p

**Figure 1:**
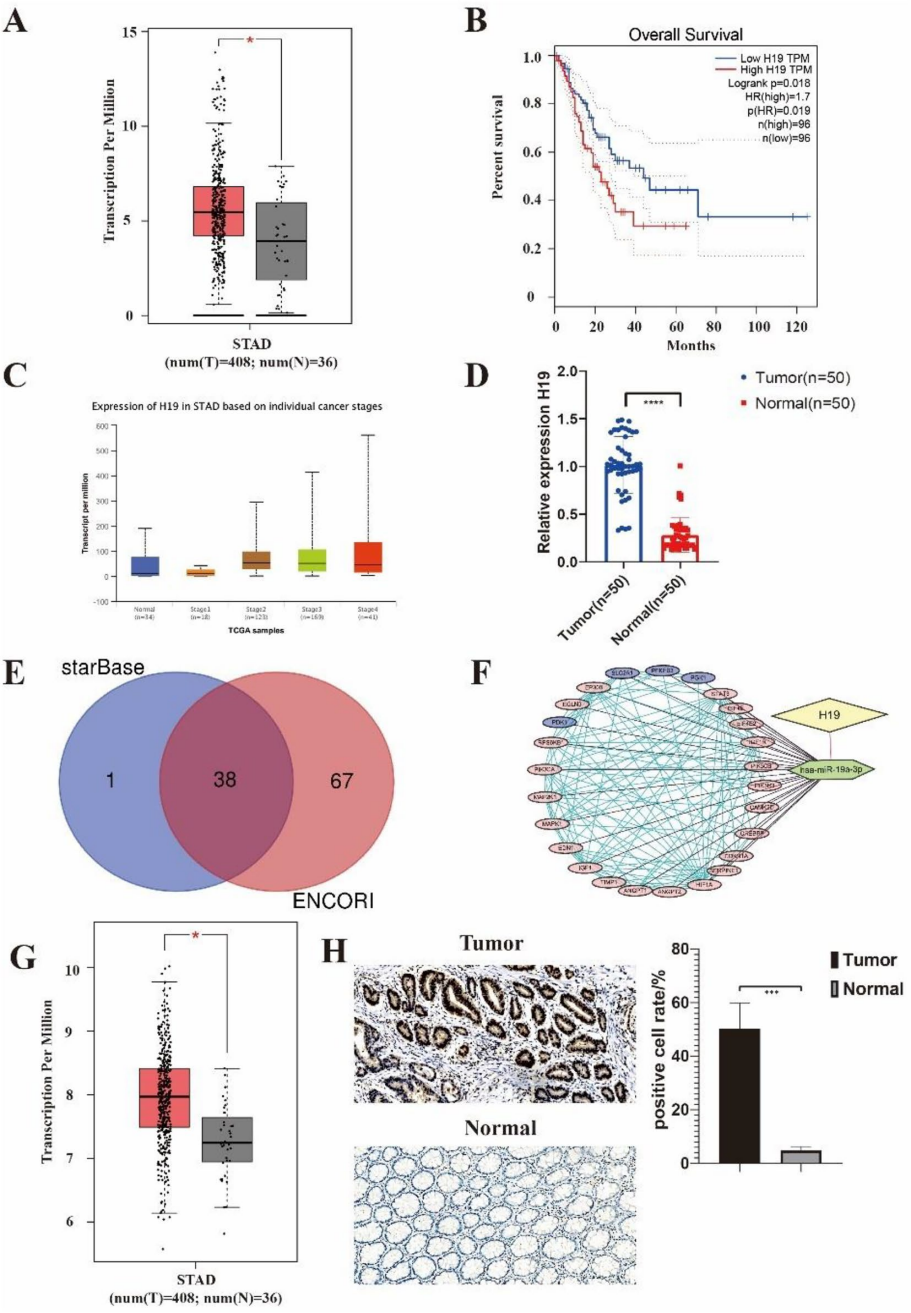
The H19 expression profiles and regulated miRNA were obtained from the public database. (**A**) H19 gene expression from the TCGA database between normal and tumor tissues derived from clinical patients with gastric cancer. (**B**) Kaplan–Meier analysis of H19 expression at low or high levels at the endpoint of overall survival rate in patients from TCGA. (**C**) Expression of H19 in different pathological stages of gastric cancer patients. (**D**) Quantifying H19 expression analysis of gastric cancer patients. (**E**) Predict the miRNAs that H19 can regulate through the database. We used two different databases (starBase and ENCORI) to predict miRNAs by H19 sequence mapping and get the cross-miRNA by Veen analysis. (**F**) We performed the target mRNA prediction separately by miRNAs we got before and further analyzed the target mRNAs by GO and KEGG. (**G**) PGK1 expression from the TCGA database between normal and tumor tissues derived from clinical patients with gastric cancer. (**H**) Immunohistochemistry showed PGK1 expression from the gastric cancer patients between normal and tumor tissues. *P < 0.05; **P < 0.01; ***P < 0.001; ****P < 0.0001.

The expression of H19 was considerably higher in GC tissue samples compared to normal samples, according to a new database visualization website of GEPIA with TCGA data (http://gepia.cancerpku.cn/) (Fig. 1A). Meanwhile, H19 expression also increased with the higher pathological stage (Fig. 1C). By using an RT-qPCR assay to compare the levels of H19 expression in 50 pairs of adjacent normal tissues and GC tissues, we discovered that the relative expression of H19 was greater in tumor tissues than in normal tissues (Fig. 1D), we were able to confirm the aforementioned findings. Using GEPIA and TCGA data, we further assessed the prognostic utility of H19 in GC. As shown in Fig. 1B, GC patients with high H19 expression had a shorter overall survival time. To forecast the miRNAs that correspond to H19, we continued to use online data (Fig. 1E) and found 38 miRNAs may be affected by H19 (Supplementary Table 1). By analyzing the data and overlapping with the previous study^20^, we discovered that four molecules that are related to glycolysis were controlled by MiR-19a-3p (Fig. 1F). Then we searched online and found the expression of PGK1 is higher in the tumor tissues than the expression in the normal tissues (Fig. 1G). After this, we further measured the expression of the aerobic glycolysis marker named PGK1 in the patients by Immunohistochemistry and also verified the result that PGK1 increased in tumor tissues than in normal tissues (Fig. 1H).

### H19 knockdown reduced glycolysis in GC

**Figure 2:**
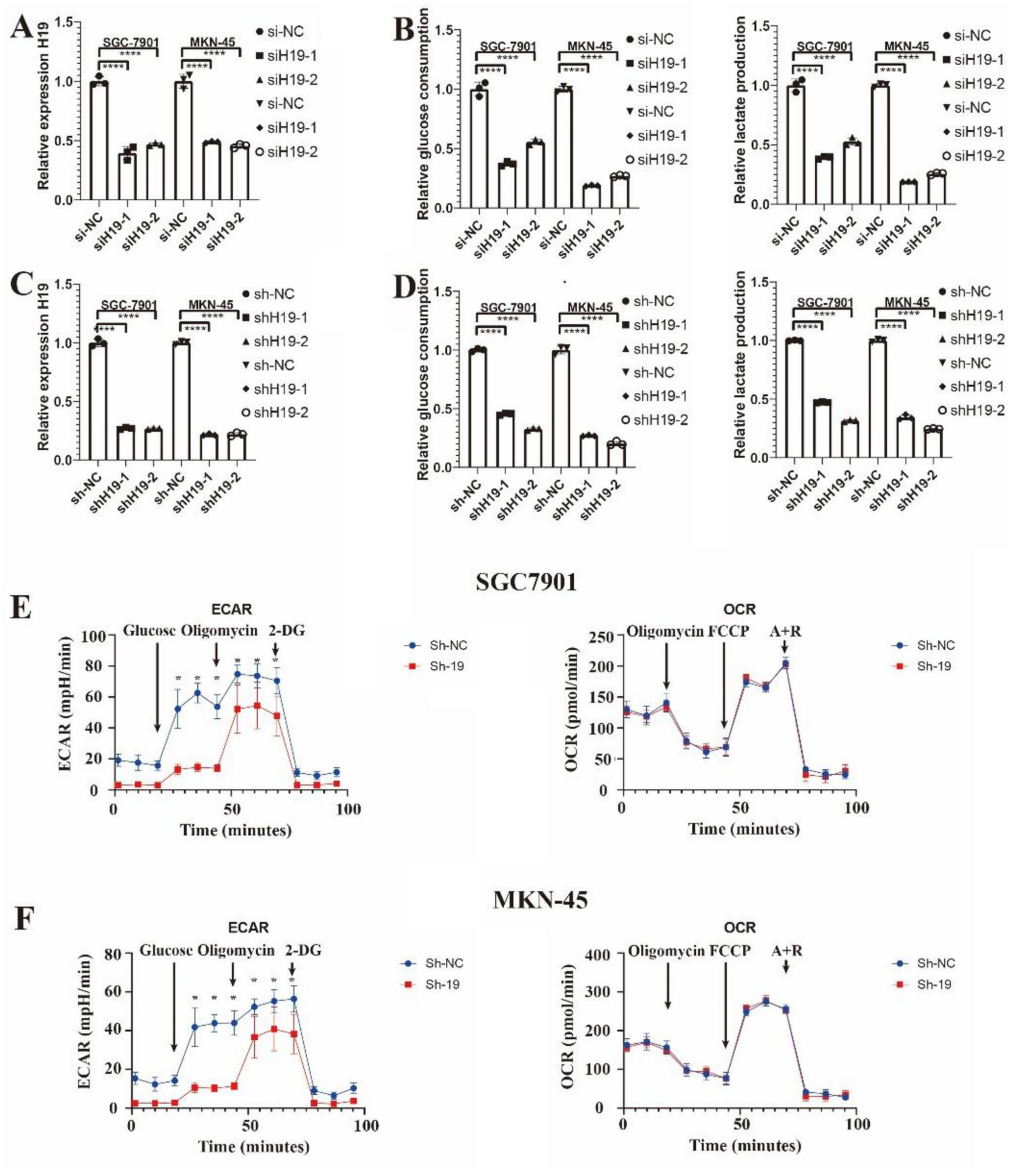
The expression of H19 is related to cellular glucose consumption, lactate production, and energy metabolism. (**A**) qPCR results of knockdown of H19 using siRNA. (**B**) The results glucose consumption and lactate production after knockdown of H19. (**C**) qPCR results of knockdown of H19 using siRNA. (**D**) The results glucose consumption and lactate production after knockdown of H19. (**E** and **F**) ECAR assays showed control and H19-knockdown in two different cells (**E**) and control, H19 with shRNA-1 or shRNA-2 SGC-7901 cells, (**F**) H19 with shRNA-1 or shRNA-2 MKN-45 cells. Results are displayed as the mean ± SD of quintuplicate measurements repeated at least three times with similar results. *P < 0.05; **P < 0.01; ***P < 0.001; ****P < 0.0001.

We postulated that H19 might be involved in the glycolysis process in GC cells given that H19 could control aerobic glycolysis in breast cancer stem cells and ovarian cancer cells^21,22^. In order to create the H19 reduction cell model, two siRNAs (siH19-1 and siH19-2) were transfected into SGC-7901 and MKN-45 cells (Fig. 2A). H19 reduction significantly decreased the amount of glucose that SGC-7901 and MKN-45 cells consumed, as shown in Fig. 2B. Additionally, we examined how suppressing H19 affected lactate production and discovered that doing so greatly reduced lactate production (Fig. 2B). In stable knockdown H19 SGC-7901 or MKN-45 cells (Fig. 2C), glucose consumption and lactate production were both significantly reduced, supporting the findings from above (Fig. 2D). In addition, we detected energy metabolism in SGC-7901 and MKN-45 cells by seahorse. Similar to our previous results, the extracellular acidification rate (ECAR) decreased significantly after knocking down H19 in SGC-7901 and MKN-45 cells, separately. Meanwhile, there is no difference in oxygen consumption rate (OCR) after H19 knockdown in two cell lines (Fig. 2E,F). These findings showed that in GC cells, H19 knockdown hindered aerobic glycolysis.

### H19 affected aerobic metabolism by PGK1

**Figure 3:**
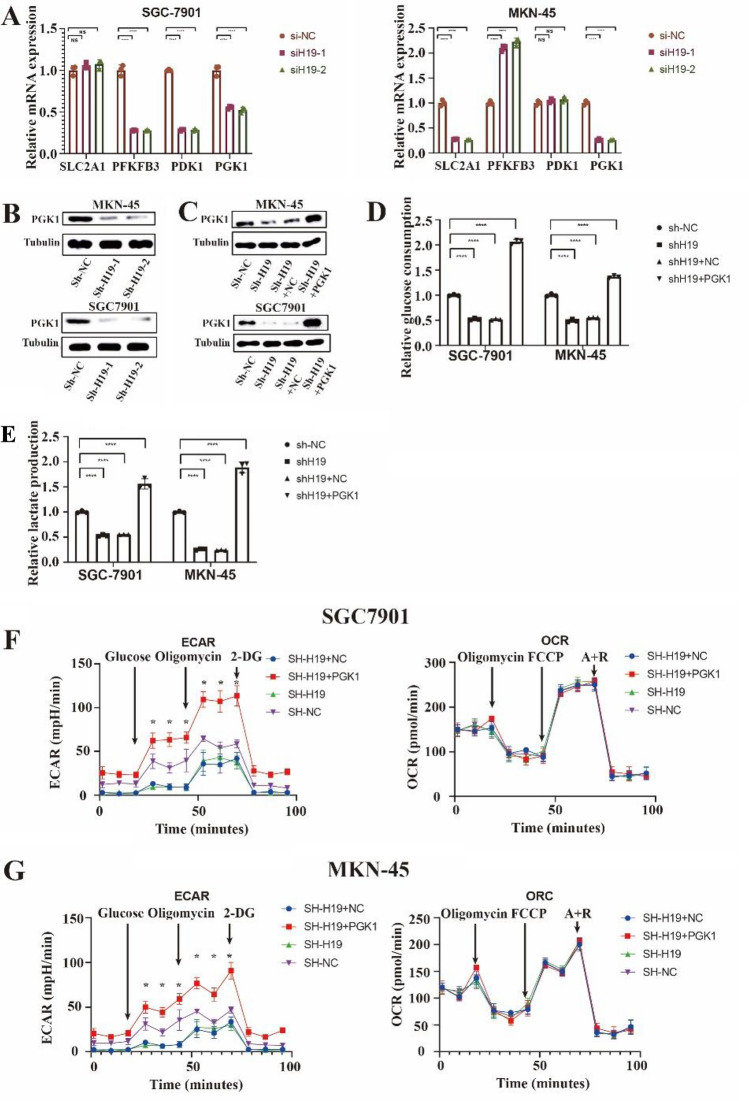
H19 knockdown inhibited aerobic glycolysis via Phosphoglycerate kinase 1 (PGK1). A Real-time quantitative PCR was used to assess the expression of genes linked to glycolysis in SGC 7901 and MKN 45 cells after transfection with siRNA negative control (siNC), H19 siRNA-1, or H19 siRNA-2. B, Western blot analysis was used to determine the amount of PGK1 protein in SGC 7901 and MKN 45 cells following transduction with shNC, shH191, or shH192. A loading limit was provided by tubulin. C, Both shH19 SGC-7901 and MKN-45 cells were transfected with the PGK1 overexpression plasmid or the negative control vector, and the PGK1 protein level was found by western blot in both cells (PGK1). A loading limit was provided by tubulin. D, E, Glucose consumption (**D**), and lactate production (**E**) were examined in sh‐H19 SGC‐7901 and MKN‐45 cells after transfection with NC or PGK1. (**F** and **G**) ECAR assays showed on control, H19-knockdown and PGK1-OE in two different cells (**F**) and control, H19 with shRNA-1 or shRNA-2 and PGK1-OE SGC-7901 cells, (**G**) H19 with shRNA-1 or shRNA-2 and PGK1-OE MKN-45 cells. Each experiment was repeated at least three times with similar results. Data are presented as the mean ± SD and analyzed by Student’s t‐test. ns, not significant; *P < 0.05; **P < 0.01; ***P < 0.001; ****P < 0.0001. SLC2A1, solute carrier family 2 member 1; PFKFB3, 6-phosphofructo-2-kinase/fructose-2,6-biphosphatase 3; PDK1, phosphoinositide‐dependent kinase‐1.

Subsequently, we detected the important molecules involved in aerobic glycolysis predicted above and found that when siRNA was used to knock down H19, only the expression of PGK1 was down-regulated in the two cell lines, and the protein expression decreased when H19 was stably knocked down by shRNA as well (Fig. 3A,B). Therefore, we wondered whether H19 regulates glucose consumption and lactate production through its effect on PGK1. We got a commercial PGK1 overexpression plasmid (PGK1) to investigate whether PGK1 contributed to H19-mediated glycolysis. Figure 3C illustrates PGK1-overexpression eliminated the impact of H19 suppression on the protein production of PGK1 in SGC-7901 and MKN-45 cells. More significantly, H19 depletion-induced reductions in glucose consumption and lactate production were corrected by PGK1 overexpression (Fig. 3D,E). Additionally, In SGC-7901 and MKN-45 cells, PGK1 overexpression also eliminated the impact of H19 suppression on energy metabolism (Fig. 3F,G), as well as ECAR was higher after PGK1 overexpression and there is no difference in OCR.

### MiR-19a3p-dependent regulation of PGK1 expression and glycolysis by H19

**Figure 4:**
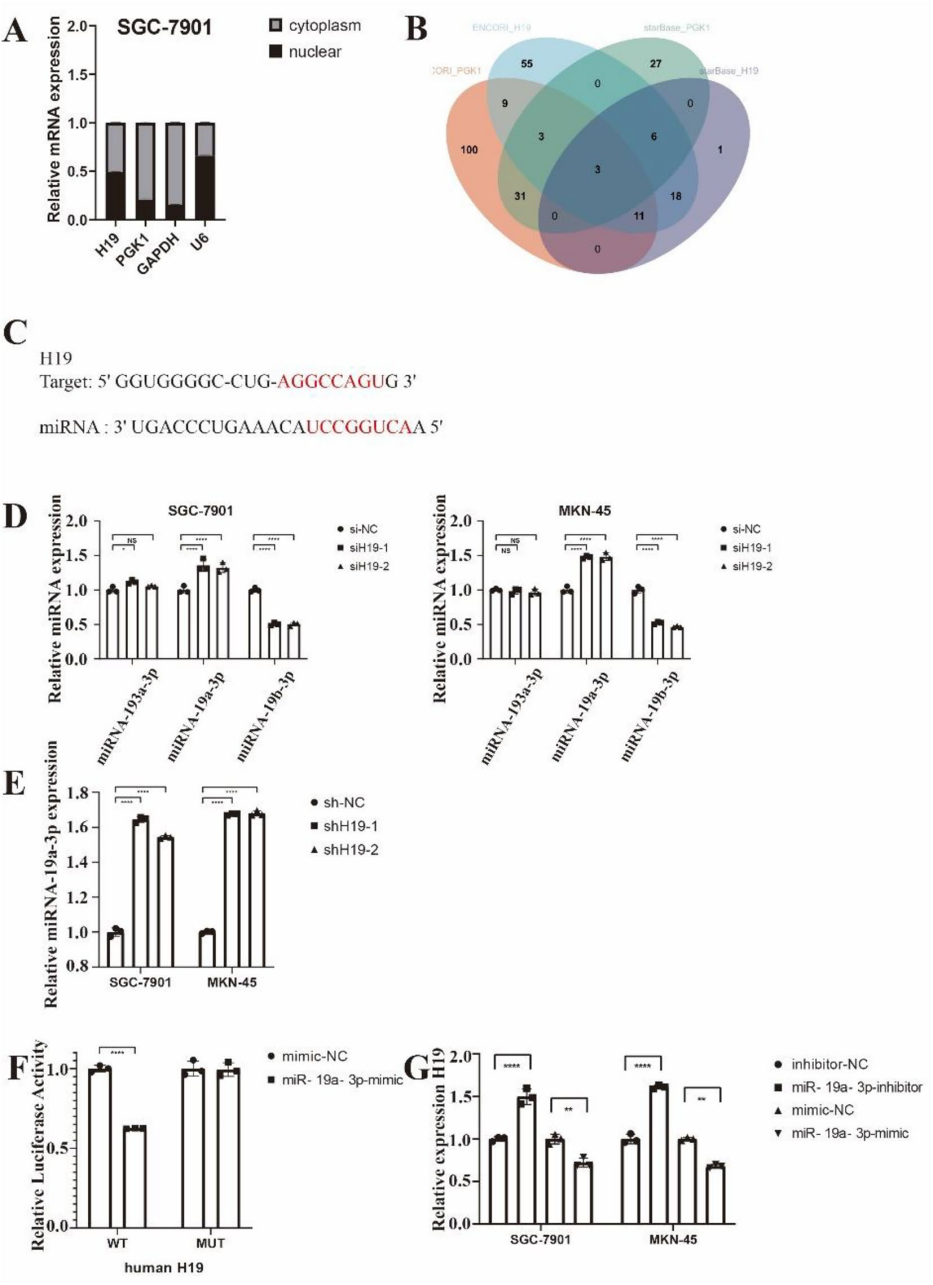
H19 sponged microRNA (miR)‐19a‐3p in gastric cancer cells. (**A**) Real-time quantitative PCR (RT-qPCR) was used to analyze nuclear and cytoplasmic separation studies in SGC-7901 cells to determine the subcellular distribution of H19 and phosphoglycerate kinase 1 (PGK1). (**B**) Two separate algorithms predicted three putative miRNAs that target both H19 and PGK1 (Starbase 3.0 and ENCORI for H19 and PGK1). (**C**) Hybridization models between H19 and miR‐19a‐3p. (**D**) After treatment with siRNA negative control (siNC), H19 siRNA-1 (siH19-1), or H19 siRNA-2 (siH19-2), levels of miR-193a3p, miR-19a3p, and miR-19b3p were measured by RT-qPCR in SGC-7901 and MKN-45 cells. (**E**) Levels of miR‐19a‐3p in the SGC‐7901 and MKN‐45 cells from the sh‐H19 or sh‐NC group were measured by RT‐qPCR. (**F**) The miR-19a-3p and WT H19 were shown to bond together by the luciferase reporter assay, but not mutant (MUT) H19. (**G**) After transfection with inhibitor negative control (NC), miR-19a3p inhibitor, mimic NC, or miR-19a3p mimic, RNA level of H19 was assessed by RT-qPCR in SGC-7901 and MKN-45 cells. Each experiment was repeated at least three times with similar results. Data are presented as the mean ± SD and analyzed by Student’s t‐test. ns, not significant; *P < 0.05; **P < 0.01; ***P < 0.001; ****P < 0.0001.

We used cytoplasmic and nuclear separation studies with RT-qPCR to ascertain the subcellular localization of H19 and PGK1 to investigate how H19 controlled PGK1 expression in GC cells. The findings revealed that PGK1 was primarily localized in the cytoplasm while H19 was dispersed throughout the nucleus (Fig. 4A). Additionally, lncATLAS's expected outcomes showed that H19 was localized in the cytoplasm and nucleus. As a result, we hypothesized that H19 might act as a ceRNA to control PGK1 translation by sponging one or more particular miRNAs. Therefore, we used the two openly accessible prediction tools starBase 3.0 (for H19 and PGK1) and ENCORI (for H19 and PGK1) to predict the potential binding miRNAs of H19 and PGK1 (for H19). Three miRNAs (miR-193a-3p, miR-19a-3p, and miR-19b-3p) were able to attach to both H19 and PGK1, as seen in Fig. 4B. After that, RT-qPCR was used to measure the levels of the three candidate miRNAs, and the findings showed that miR-19a-3p expression was considerably higher in H19 knockdown cells than it was in SGC-7901 and MKN-45 cells (Fig. 4D). Interestingly, in our experiments, we found that the expression of miR-19b-3p decreased after knocking down H19. Subsequently we used the miR-19b-3p inhibitor, but we found that miR-19b-3p could not rescue the decreased energy metabolism and PGK1 expression caused by H19 knockdown (Supplementary Fig. 1). Additionally, when compared to the shNC group, the expression of miR-19a-3p in SGC-7901 and MKN-45 cells from the shH19 group was noticeably elevated (Fig. 4E). We displayed the hybridization models between H19 and miR-19a-3p according to the predicted matching sequence (Fig. 4C and F). We then performed luciferase reporter experiments to see if miR-19a-3p could directly control H19. The luciferase activity of the pmirGLOH19WT vector was reduced but not the pmirGLOH19MUT vector when miR-19a-3p was overexpressed (Fig. 4F). Additionally, the findings of RT-qPCR showed that H19 levels in SGC-7901 and MKN-45 cells were significantly higher when miR-19a-3p was downregulated than when it was overexpressed (Fig. 4G).

**Figure 5:**
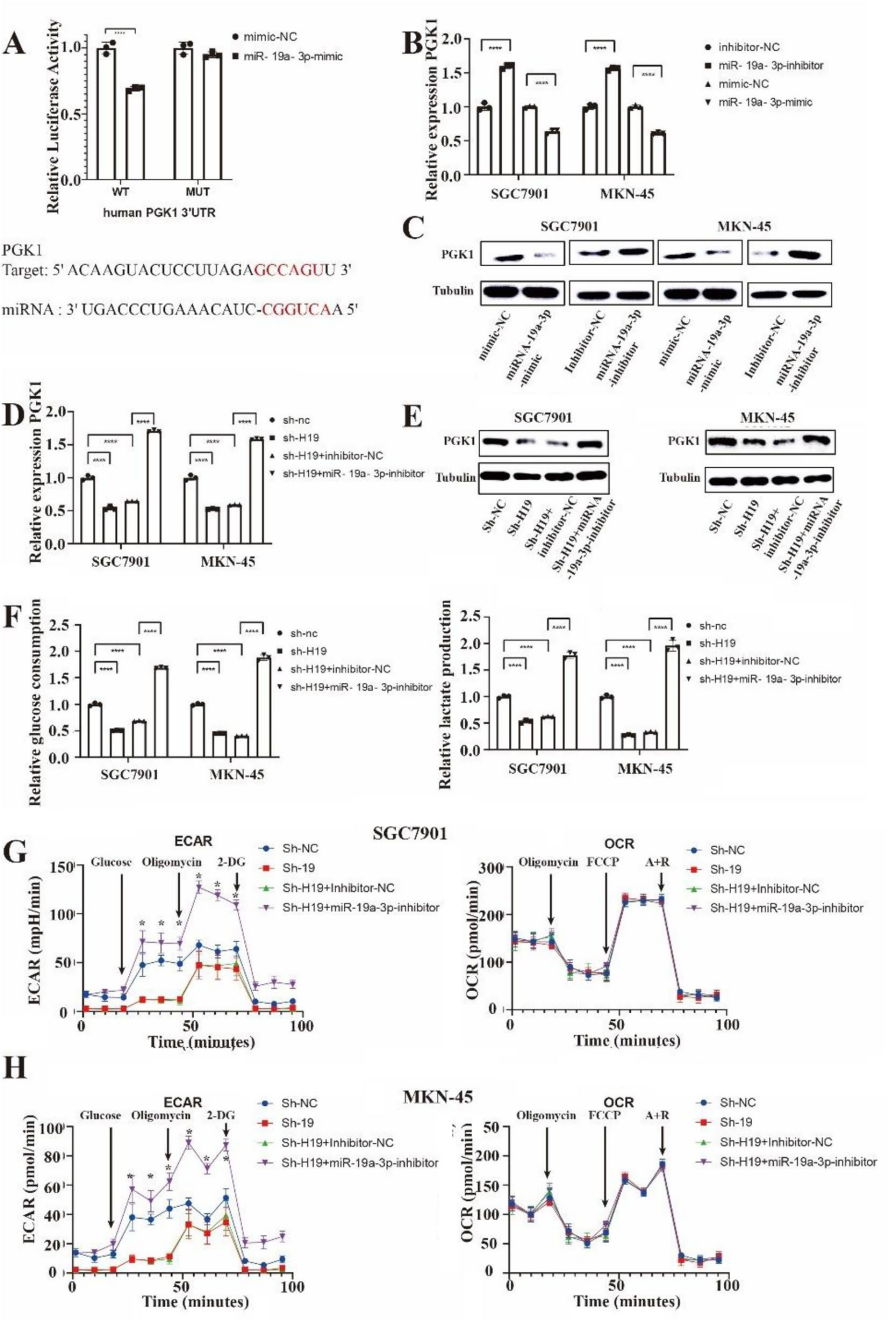
H19 regulated phosphoglycerate kinase 1 (PGK1) expression and glycolysis through microRNA (miR)‐19a‐3p. (**A**) Hybridization models between PGK1 and miR‐19a‐3p and luciferase reporter assay showed the binding of miR‐19a‐3p and WT PGK1 3′‐UTR but not mutant (MUT) PGK1 3′‐UTR. (**C**, **D**) mRNA (**B**), and protein (**C**) levels of PGK1 were measured in both SGC‐7901 and MKN‐45 cells after transfection with inhibitor negative control (NC), miR‐19a‐3p inhibitor, mimic NC, or miR‐19a‐3p mimic. Tubulin served as a loading control. (**E**, **F**) mRNA (**D**), and protein (**E**) levels of PGK1 were measured in both sh‐H19 SGC‐7901 and MKN‐45 cells after transfection with inhibitor NC or miR‐19a‐3p inhibitor. Tubulin served as a loading control. (**F**) Glucose consumption and lactate production were examined in both sh‐H19 SGC‐7901 and MKN‐45 cells after transfection with inhibitor NC or miR‐19a‐3p inhibitor. (**G** and **H**) ECAR assays showed on control, H19-knockdown and PGK1-OE in two different cells (**G**) and control, H19 with shRNA-1 or shRNA-2, inhibitor NC and miR‐19a‐3p inhibitor in SGC-7901 cells, (**H**) H19 with shRNA-1 or shRNA-2, inhibitor NC and miR‐19a‐3p inhibitor in MKN-45 cells. Each experiment was repeated at least three times with similar results. Data are presented as the mean ± SD and analyzed by Student’s t‐test. ns, not significant; *P < 0.05; **P < 0.01; ***P < 0.001; ****P < 0.0001.

Figure 5A displays the hybridization models between PGK1 and miR-19a-3p. The direct association between PGK1 and miR-19a-3p was verified by luciferase reporter assays. The pmirGLOPGK1WT vector's luciferase activity was decreased by the miR-19a-3p mimic, but the pmirGLOPGK1MUT vector's activity was not decreased (Fig. 5A). In SGC-7901 and MKN-45 cells, miR-19a-3p overexpression decreased while miR-19a-3p inhibition increased PGK1 mRNA and protein production (Figs. 5B,C). The rescue trials were also conducted by us. According to the findings, in the steady knockdown of H19 SGC-7901 and MKN-45 cells, miR-19a-3p inhibitor also reversed the declines in PGK1 mRNA and protein expression (Figs. 5D,E). In SGC-7901 and MKN-45 cells, miR-19a-3p inhibition also eliminated the impact of H19 suppression on glucose consumption, lactate production, and energy metabolism (Fig. 5F–H). ECAR was higher after miR-19a-3p inhibition and there is no difference in OCR. According to these findings, H19 might influence the production of PGK1 and glycolysis by sponging miR-19a-3p.

### H19 knockdown reduced GC cell proliferation through the miR19a3p/PGK1 pathway

**Figure 6:**
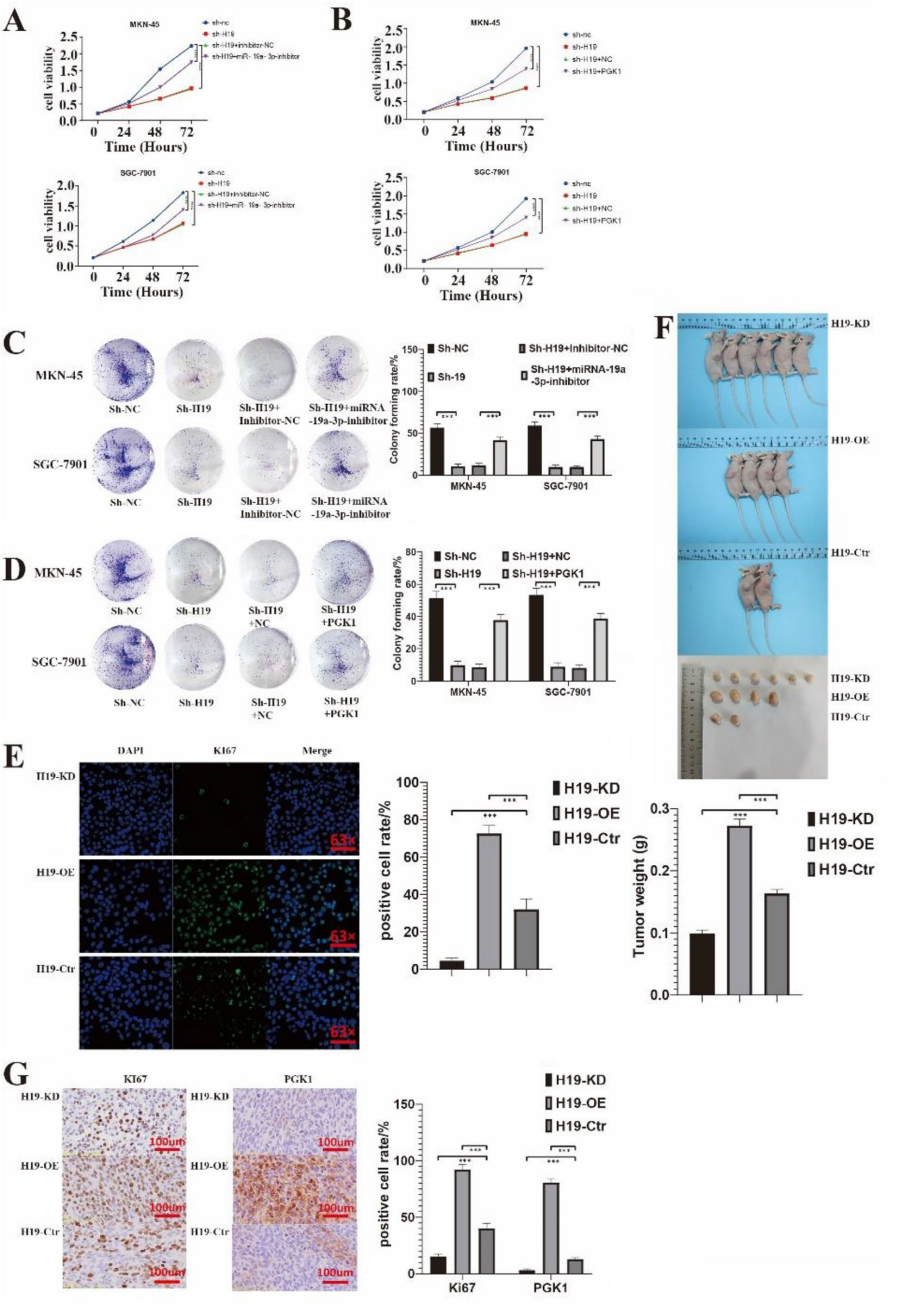
H19 knockdown suppressed gastric cancer cell proliferation through the microRNA (miR)‐19a‐3p/phosphoglycerate kinase 1 (PGK1) axis. (**A**) MTT was used to determine the cell viability of shH19 SGC 7901 and MKN 45 cells after transfection with miR-19a-3p inhibitor. (**B**) MTT was used to determine the cell viability of shH19 SGC 7901 and MKN 45 cells after transfection with PGK1 overexpression plasmid. (**C**, **D**) Colony formation assay of sh‐H19 SGC‐7901 and MKN‐45 cells after transfection with miR‐19a‐3p inhibitor (**C**) or PGK1 overexpression plasmid (**D**). (**E**) Ki-67 immunofluorescence images of control, H19 knockdown, and H19 overexpression in SGC‐7901 cells. (**F**) Subcutaneous injections of H19 control, H19 OE, and H19 KD (SGC-7901 cells, knock down by shRNA-1 to H19) were administered to nude mice. To collect the xenograft tumors, mice were put to death after 7 weeks. All xenograft growth images are displayed. (**G**) IHC of representative xenograft tumors from different treatments. Each experiment was repeated at least three times with similar results. Data are presented as the mean ± SD and analyzed by Student’s t‐test. ns, not significant; *P < 0.05; **P < 0.01; ***P < 0.001; ****P < 0.0001.

Notably, H19 has been implicated in several mechanisms that increase the proliferation of cancer cells^23–25^. Here, we looked into how H19 knockdown affected the growth of GC cells and examined the underlying molecular mechanisms. The MTT assay revealed that H19 knockdown greatly reduced the rate of GC cell proliferation (Fig. 6A,B). Clone assay was used to further corroborate these findings (Fig. 6C,D, and Supplementary Fig. 2). We next investigated whether H19 was involved in GC cell growth via the miR-19a-3p/PGK1 axis given that H19 modulated glycolysis by regulating the expression of PGK1 in a miR-19a-3p-dependent fashion. Rescue studies confirmed that miR-19a-3p reduction and PGK1 overexpression reversed the impact of H19 knockdown on GC cell proliferation rate and colony formation (Fig. 6). Ki67 staining significantly decreased as a result of immunofluorescence analyses, indicating knockdown H19 was preventing proliferation. At the same time, the rescue also was verified (Fig. 6E). Tumor volumes were different in overexpression H19, knockdown H19, and control (Fig. 6F). As well as immunohistochemistry analyses showed a significant decrease in Ki67 and PGK1 staining, suggesting that the reduction of PGK1 by H19 was inhibiting proliferation (Fig. 6G).

## Discussion

H19 has been found to play a key role in the regulation of GC tumorigenesis and development. For instance, in GC, upregulated H19 promoted cell growth and metastasis through the miR-223p/Snail1 signaling pathway and was linked with lymph node metastasis and TNM stage of GC patients^26^. According to research, miR-675 from H19 promotes GC cell growth and invasion via RUNX1^27^. Here, the results of our research were in line with earlier reports that H19 was highly expressed in GC tissues and was connected to patients with GC's poor prognosis. H19 knockdown was discovered to substantially inhibit tumor growth in vivo using a nude mouse xenograft tumor model. More significantly, we discovered that H19 influenced GC cell growth and aerobic glycolysis by sponging miR-19a-3p to activate PGK1 expression.

H19 was involved in aerobic glycolysis in cancer cells, according to earlier research. Under hypoxic conditions, the H19/let-7/HIF1 signaling-mediated PDK1 could control glycolysis and further add to breast cancer stem cell maintenance. In SKOV3 and A2780 cells treated with ginsenoside 20(S)-Rg3, H19 overexpression in ovarian cancer raised glucose consumption, lactate production, and PKM2 expression^22^. The aforementioned studies demonstrated the significant functions of H19 in the control of aerobic glycolysis in cancers, but little is known about how H19 affects aerobic glycolysis in GC. In this research, we found that H19 knockdown reduced the amount of glucose consumed and the amount of lactate produced by GC cells. Additionally, H19 levels in GC cells were favorably correlated with PGK1 mRNA and protein levels. Significantly, Elevated PGK1 was substantially linked to both advanced TNM stage in breast and esophageal cancers as well as short overall survival (OS) in cancers of the breast, liver, lung, stomach, and esophagus. Additionally, PGK1 was a hazard factor for short OS in stomach, lung, and liver cancer^28^. The previous research showed that PGK1 can promote the proliferation of cancer by turning on the AKT/mTOR pathway in non-small-cell lung cancer^19^. Therefore, we concluded that H19 controlled PGK1 and engaged in GC aerobic glycolysis. Our findings showed that PGK1 overexpression restored the reductions in glucose uptake and lactate generation caused by H19 depletion in SGC-7901 and MKN-45 cells. Ablation of PDK1 prevented H19-mediated glycolysis in breast cancer stem cells, and H19 knockdown reduced PDK1 expression through the let-7/HIF-1 axis in hypoxia. These results indicate that H19 modulated glycolysis through the let-7/HIF-1/PDK1 axis^21^. Our findings from the current research indicated that H19 has a significant impact on GC aerobic glycolysis in the presence of abundant oxygen and is PGK1-dependent. The functions of H19 in controlling aerobic glycolysis in GC cells under hypoxic circumstances are not fully understood. To find the solution to this, more research is necessary.

It is well known that lncRNAs' subcellular location significantly impacts how well they perform biologically^29^. Our findings in this case demonstrated the colocalization of PGK1 and H19 in the cytosol. Consequently, we assumed that H19 might act as a ceRNA to control PGK1 translation by sponging miRNAs. In GC cells, there was a subsequent negative association between miR-19a-3p and H19 or PGK1. Notably, miR-19a-3p acted as a tumor suppressor in a variety of malignant tumors, including colorectal cancer, prostate cancer, and pancreatic adenocarcinoma^30–32^. Here, in vitro luciferase experiments showed that miR-19a-3p had direct targets in both H19 and PGK1. The impact of H19 suppression on PGK1 expression, glucose consumption, lactate production, and cell proliferation in GC was also eliminated by miR-19a-3p inhibition. According to the aforementioned findings, H19 controlled glycolysis and cell growth via the miR-19a-3p /PGK1 pathway.

In conclusion, our findings showed that H19 plays a novel function in modulating the aerobic glycolysis and proliferation of GC. Additionally, H19 worked through the miR-19a-3p /PGK1 pathway to influence aerobic glycolysis and cell proliferation in GC cells.

### Supplementary Information

Supplementary Information 1.

Supplementary Information 2.

Supplementary Information 3.

Supplementary Information 4.

Supplementary Information 5.

Supplementary Information 6.

Supplementary Information 7.

Supplementary Information 8.

Supplementary Information 9.

Supplementary Information 10.

Supplementary Information 11.

Supplementary Information 12.

Supplementary Information 13.

Supplementary Information 14.

Supplementary Information 15.

Supplementary Information 16.

Supplementary Information 17.

Supplementary Information 18.

Supplementary Information 19.

Supplementary Information 20.

Supplementary Information 21.

Supplementary Information 22.

## Data Availability

All data generated or analyzed during this study are included in this published article.
