# Supplementary figures and images for: H19 encourages aerobic glycolysis and cell growth in gastric cancer cells through the axis of microRNA-19a-3p and phosphoglycerate kinase 1
Source: Sci Rep. 2023 Oct 11;13:17181. doi: 10.1038/s41598-023-43744-0 (PMC10567772; doi:10.1038/s41598-023-43744-0)

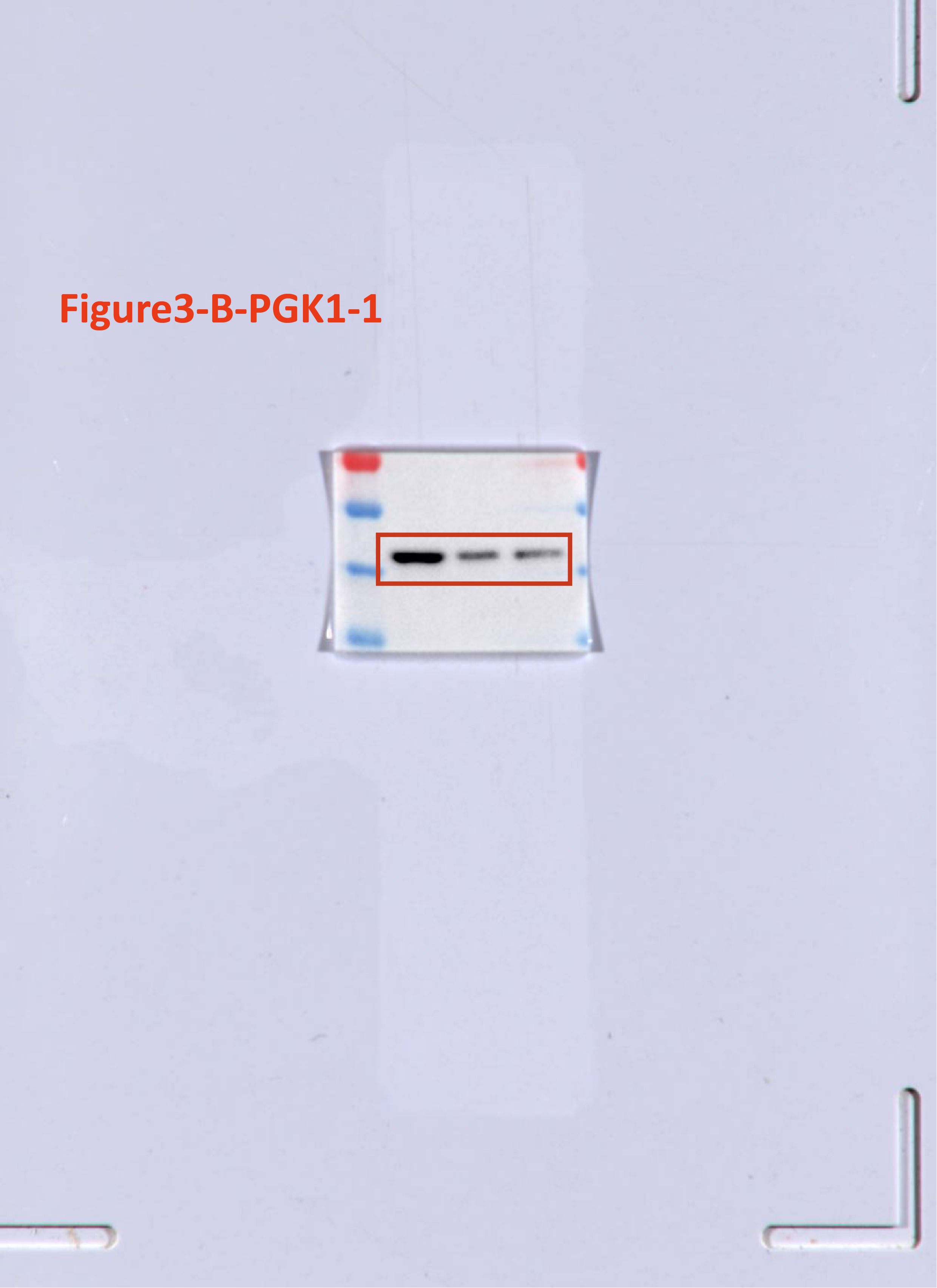

Supplement: Supplementary file 1 — Supplementary Information 1. [file 41598_2023_43744_MOESM1_ESM.jpg]

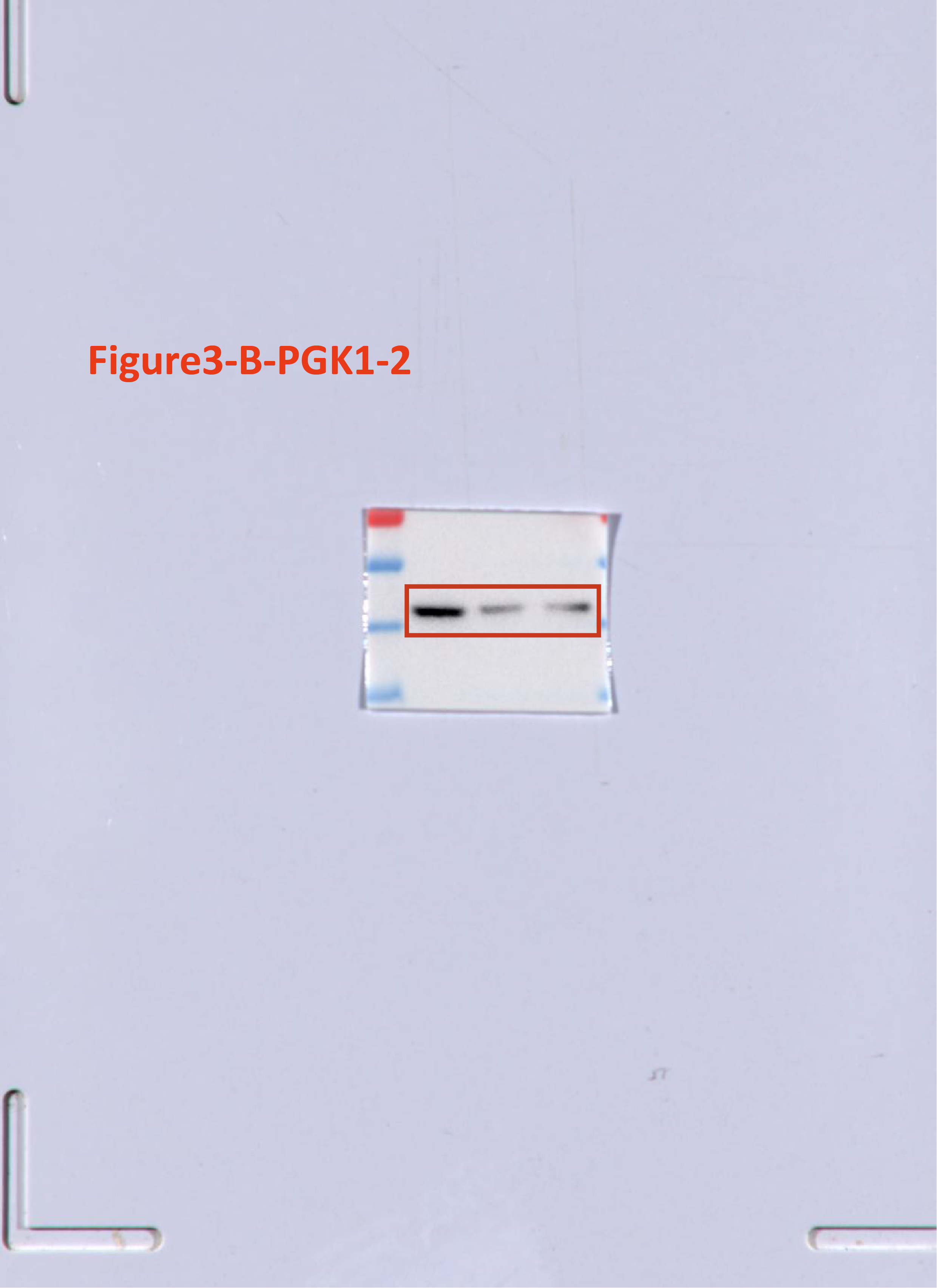

Supplement: Supplementary file 2 — Supplementary Information 2. [file 41598_2023_43744_MOESM2_ESM.jpg]

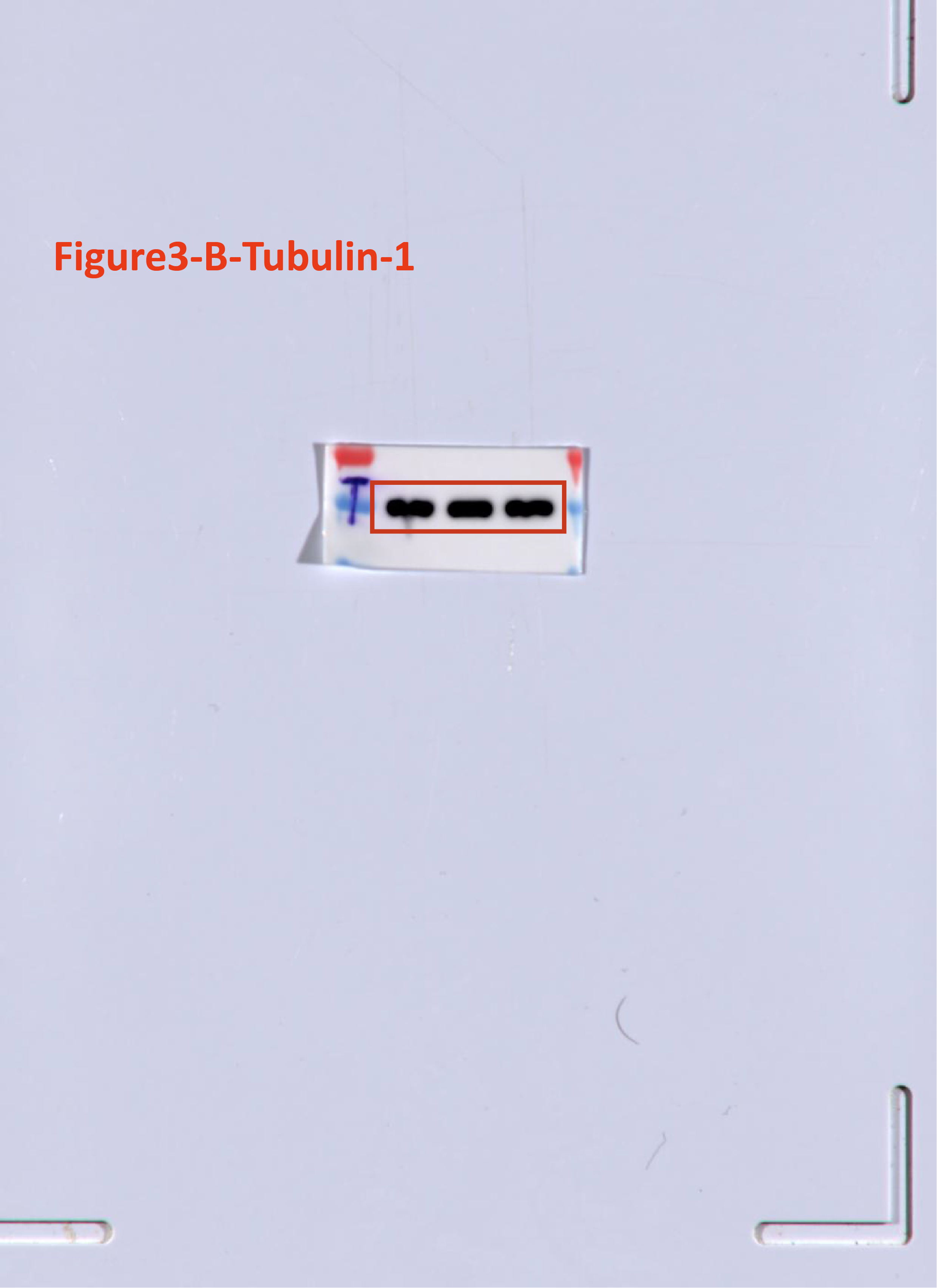

Supplement: Supplementary file 3 — Supplementary Information 3. [file 41598_2023_43744_MOESM3_ESM.jpg]

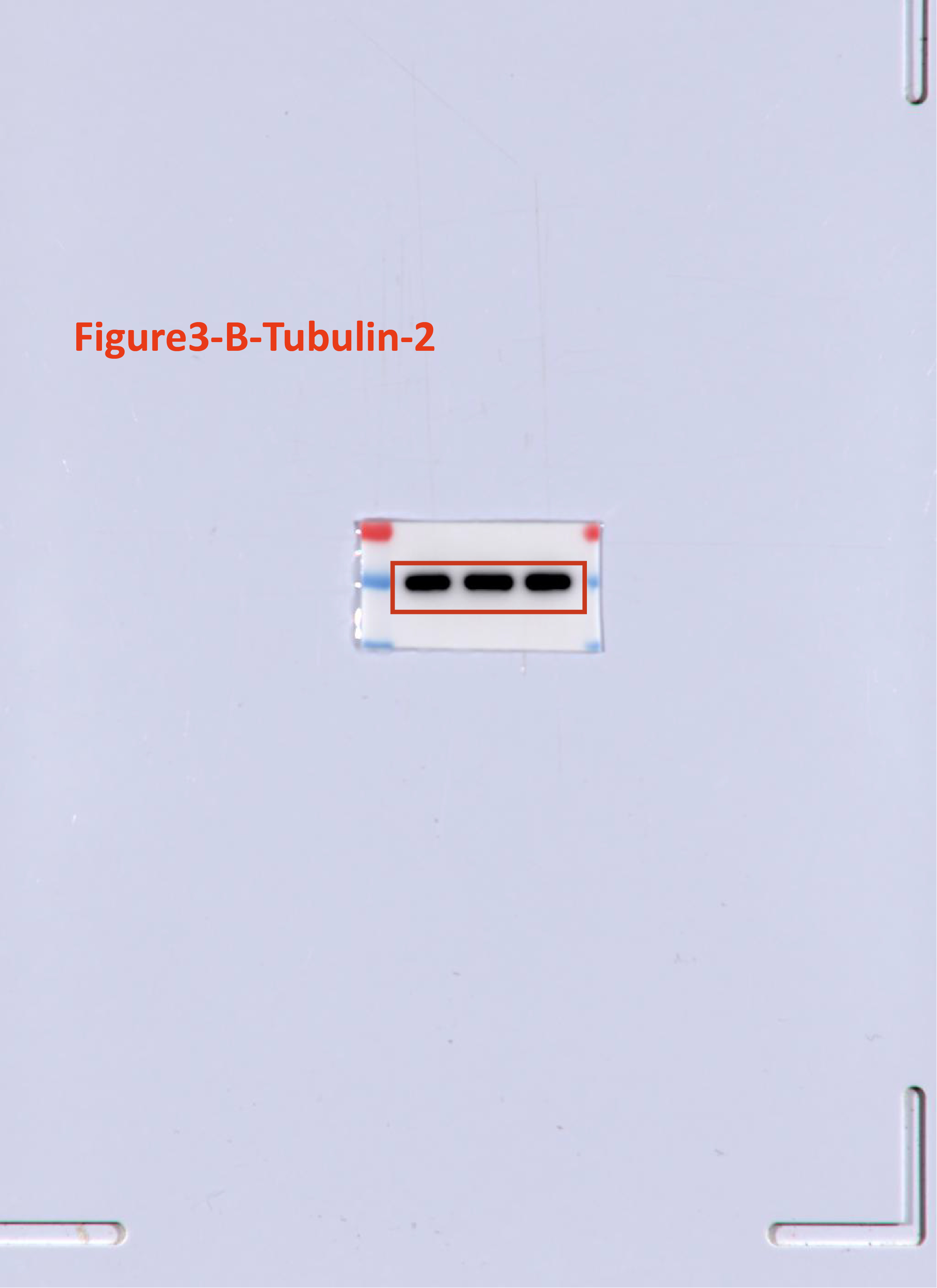

Supplement: Supplementary file 4 — Supplementary Information 4. [file 41598_2023_43744_MOESM4_ESM.jpg]

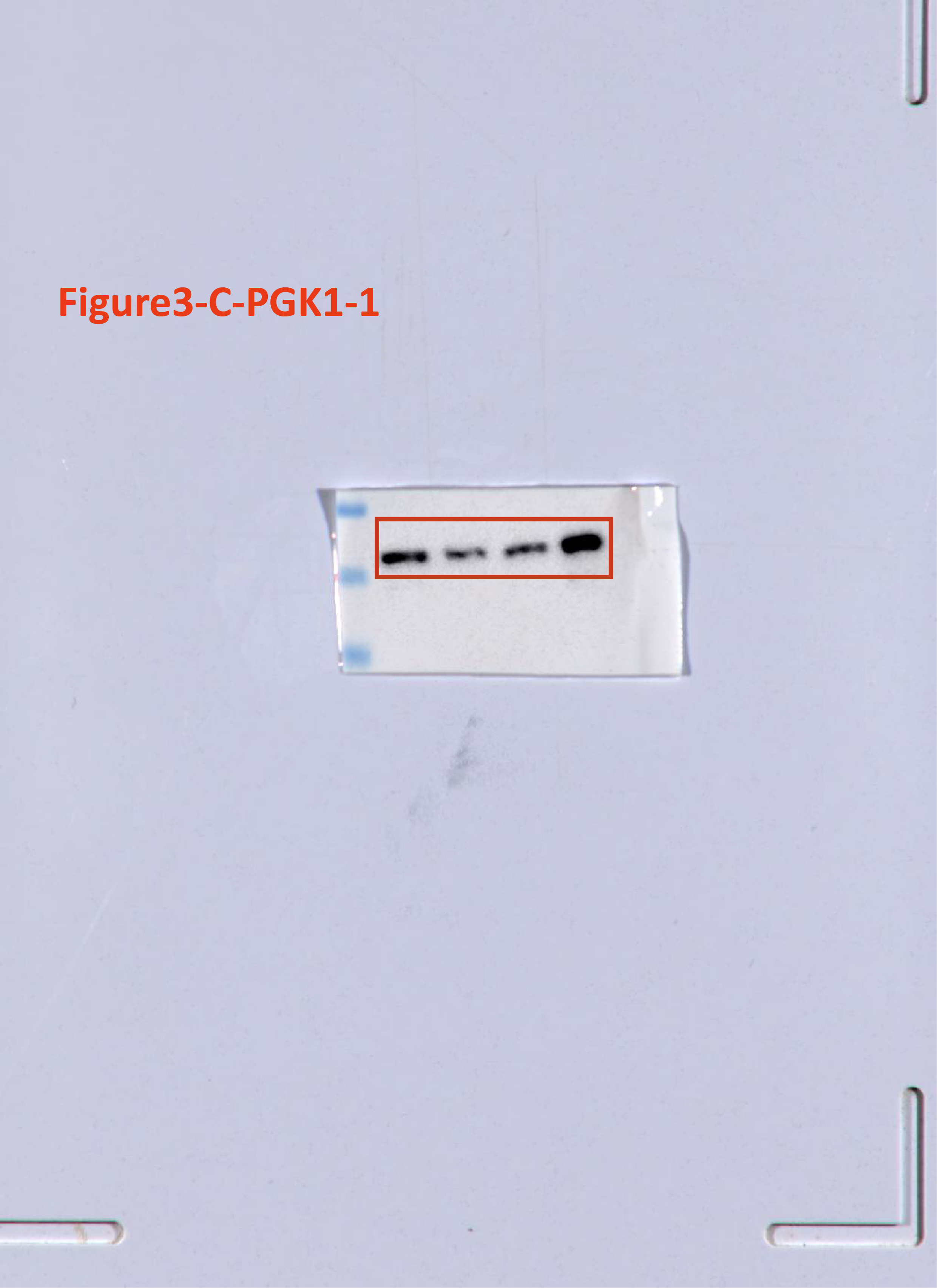

Supplement: Supplementary file 5 — Supplementary Information 5. [file 41598_2023_43744_MOESM5_ESM.jpg]

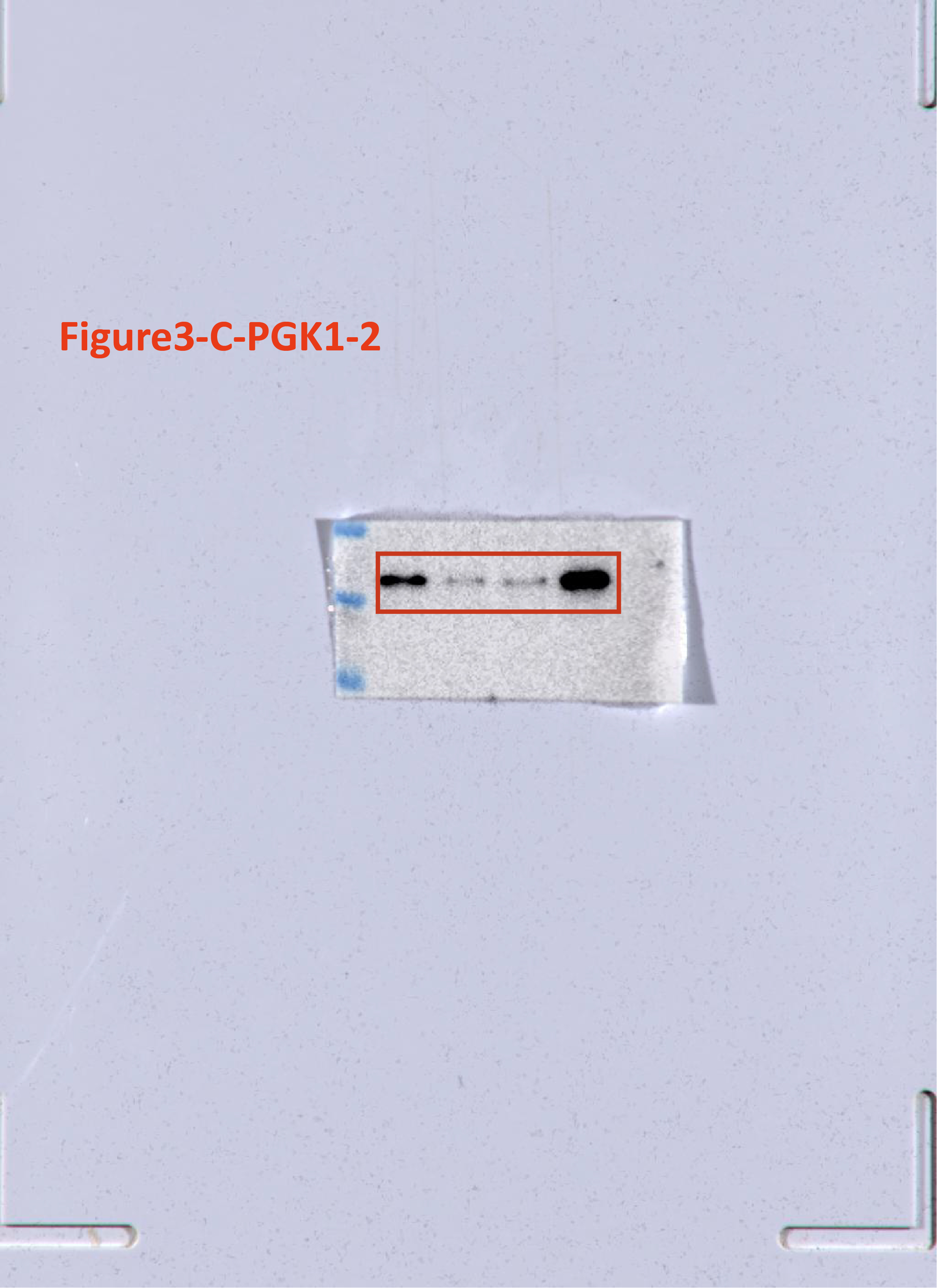

Supplement: Supplementary file 6 — Supplementary Information 6. [file 41598_2023_43744_MOESM6_ESM.jpg]

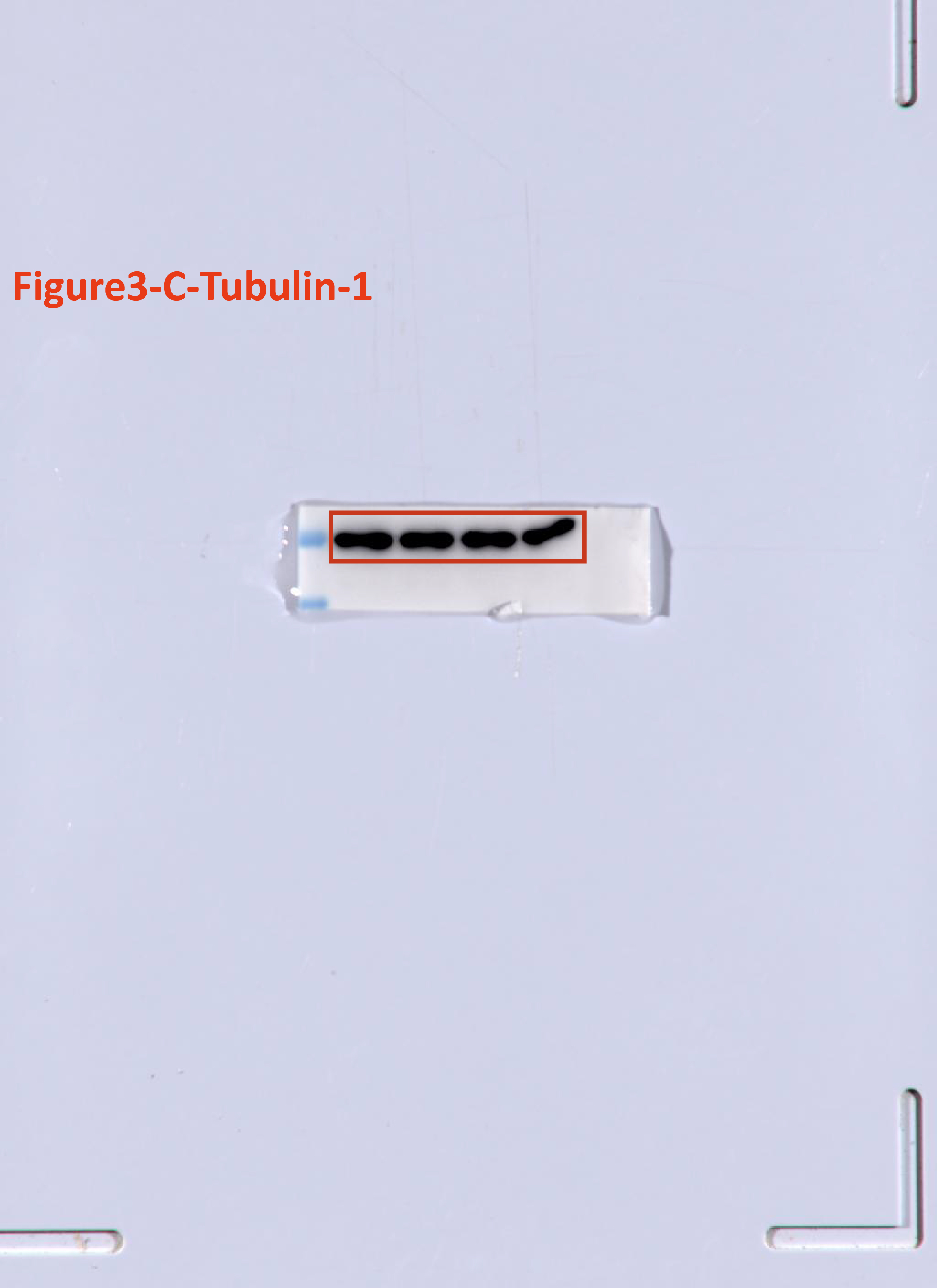

Supplement: Supplementary file 7 — Supplementary Information 7. [file 41598_2023_43744_MOESM7_ESM.jpg]

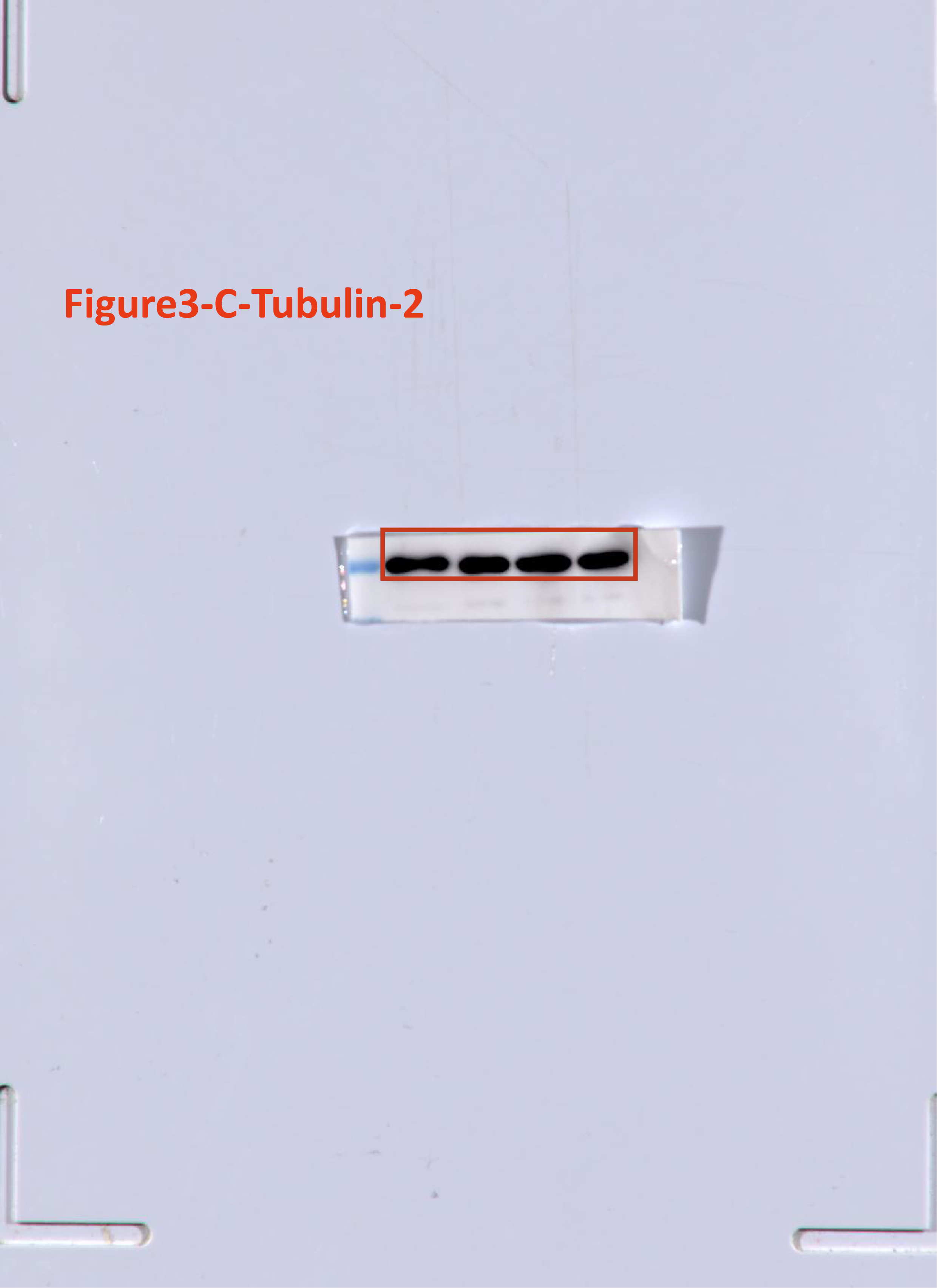

Supplement: Supplementary file 8 — Supplementary Information 8. [file 41598_2023_43744_MOESM8_ESM.jpg]

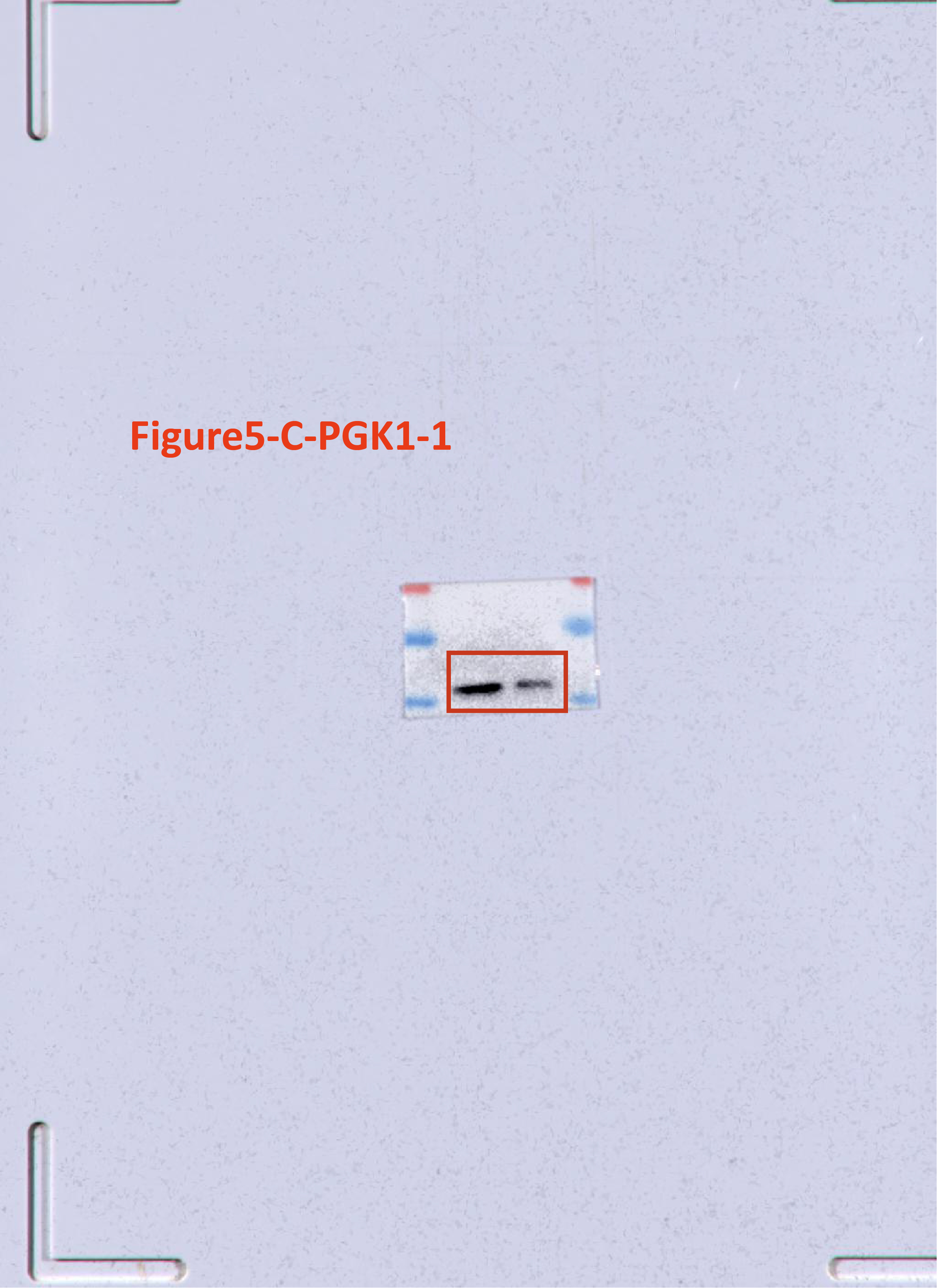

Supplement: Supplementary file 9 — Supplementary Information 9. [file 41598_2023_43744_MOESM9_ESM.jpg]

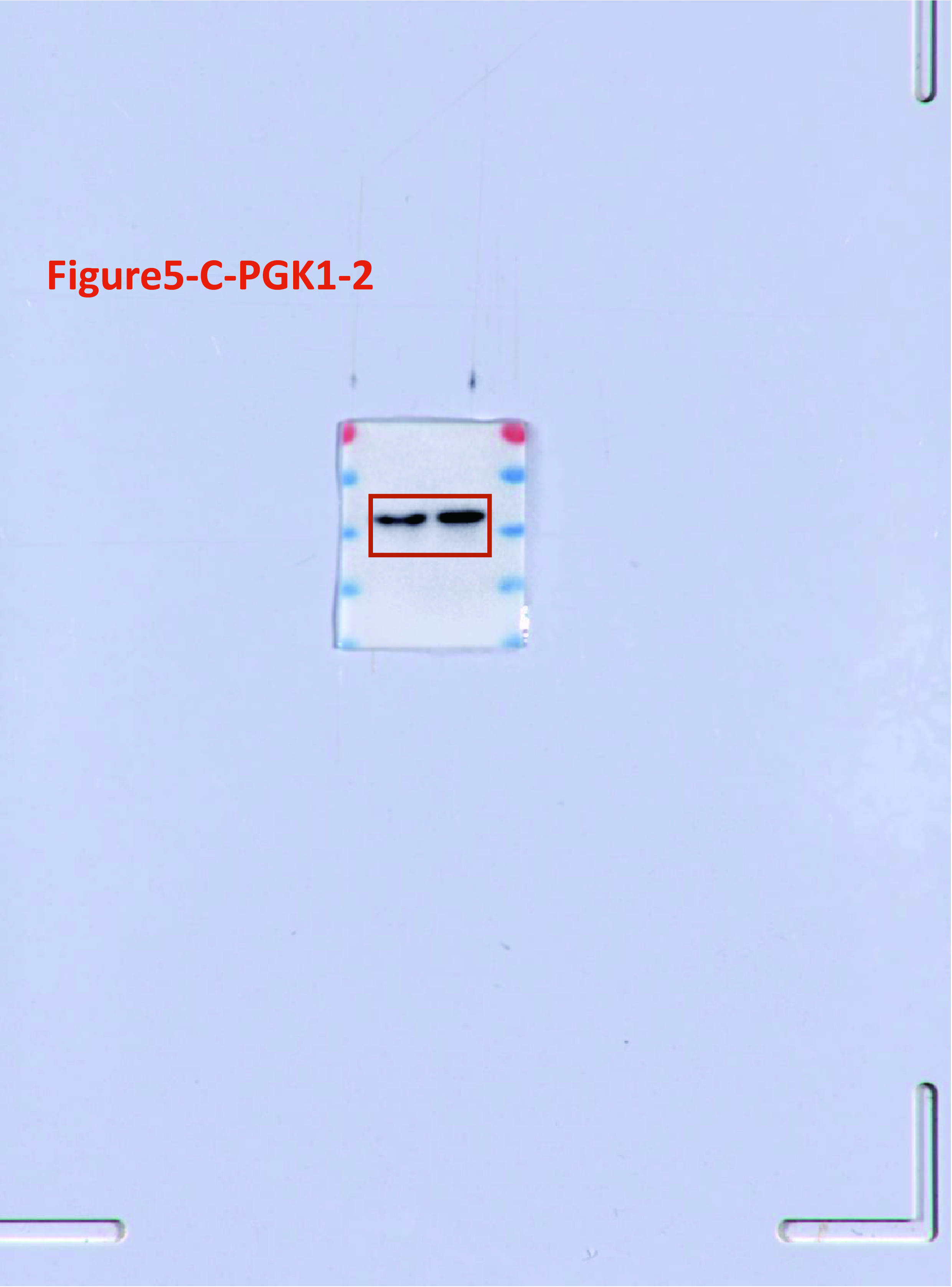

Supplement: Supplementary file 10 — Supplementary Information 10. [file 41598_2023_43744_MOESM10_ESM.jpg]

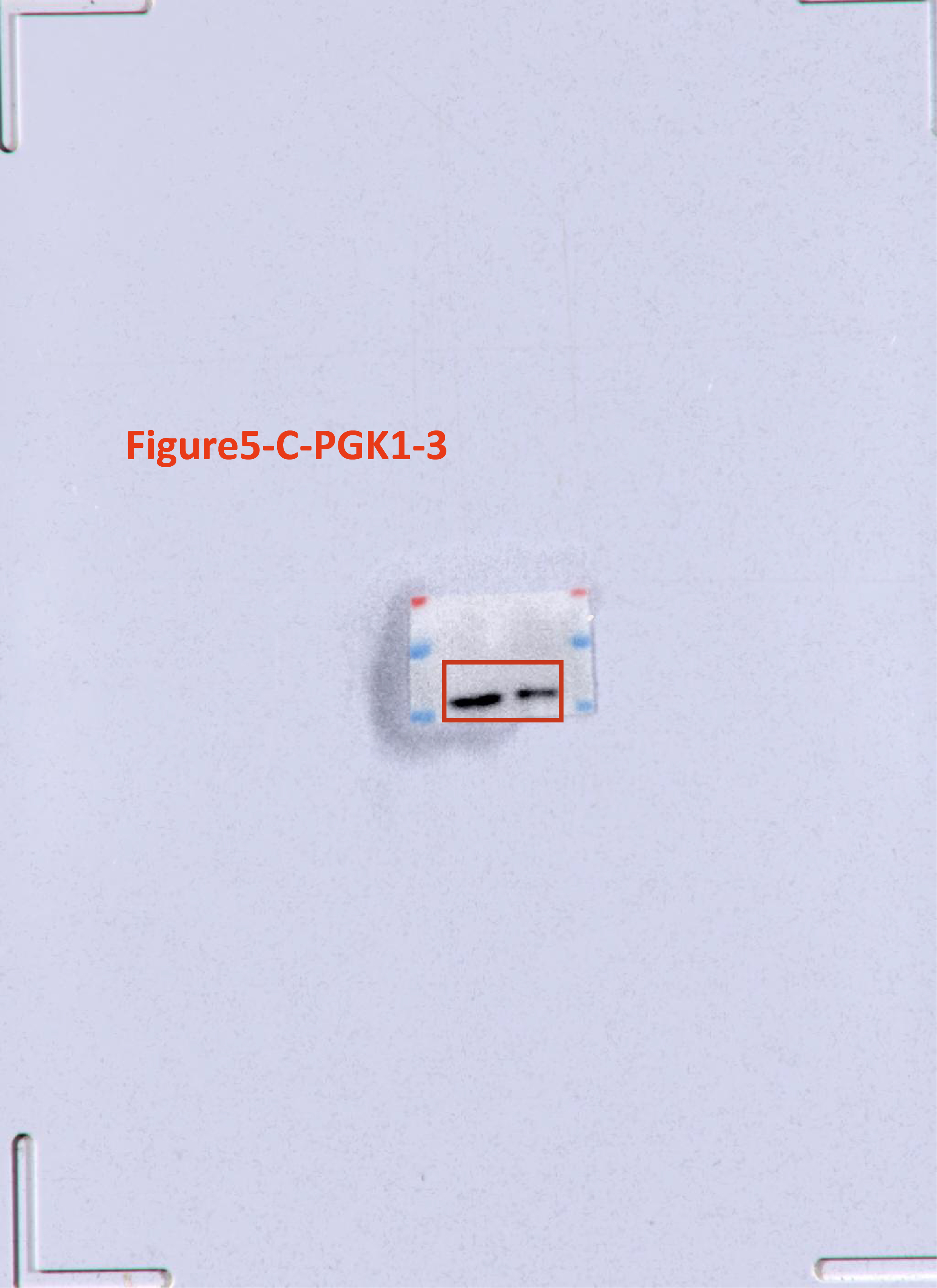

Supplement: Supplementary file 11 — Supplementary Information 11. [file 41598_2023_43744_MOESM11_ESM.jpg]

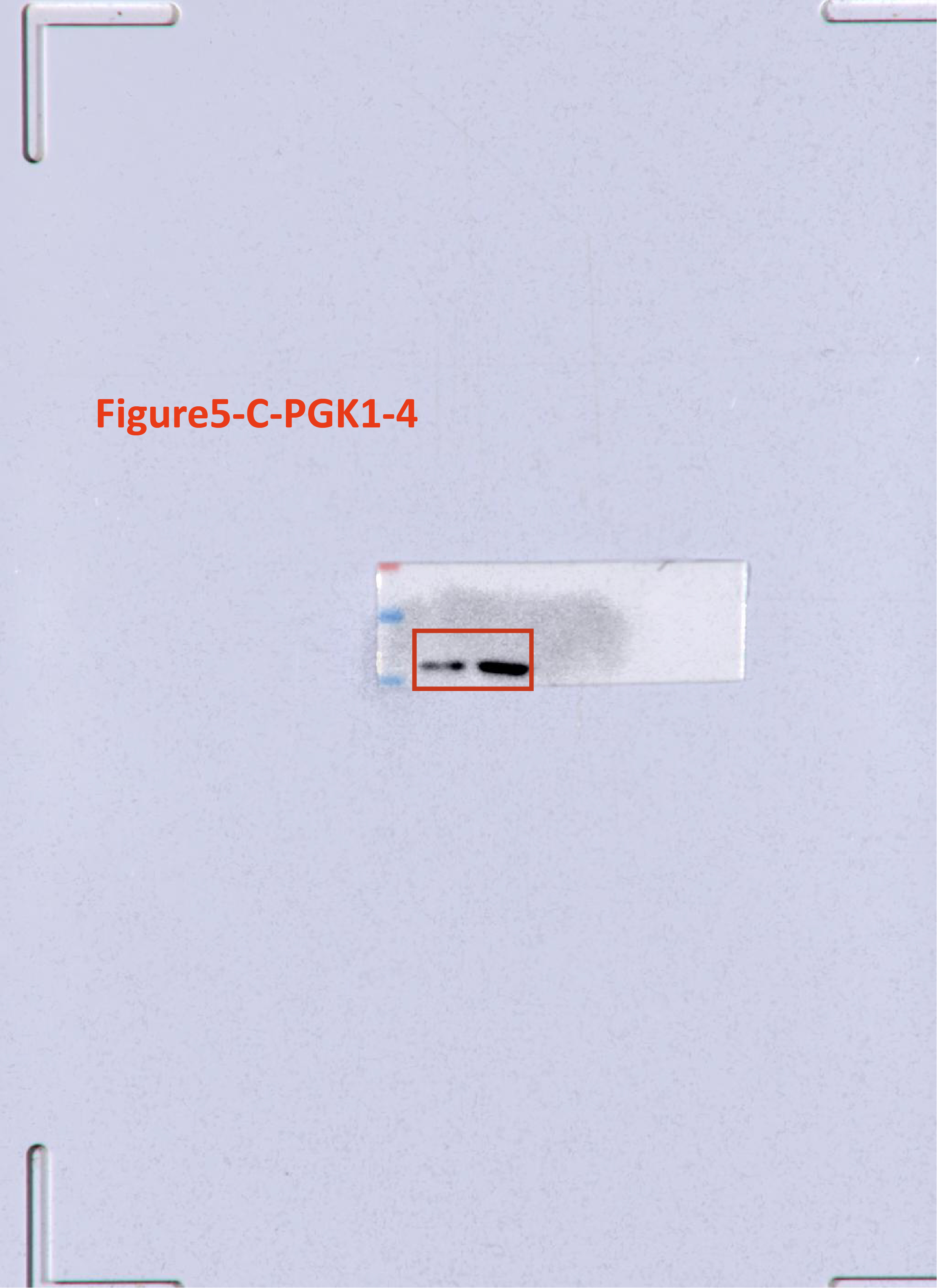

Supplement: Supplementary file 12 — Supplementary Information 12. [file 41598_2023_43744_MOESM12_ESM.jpg]

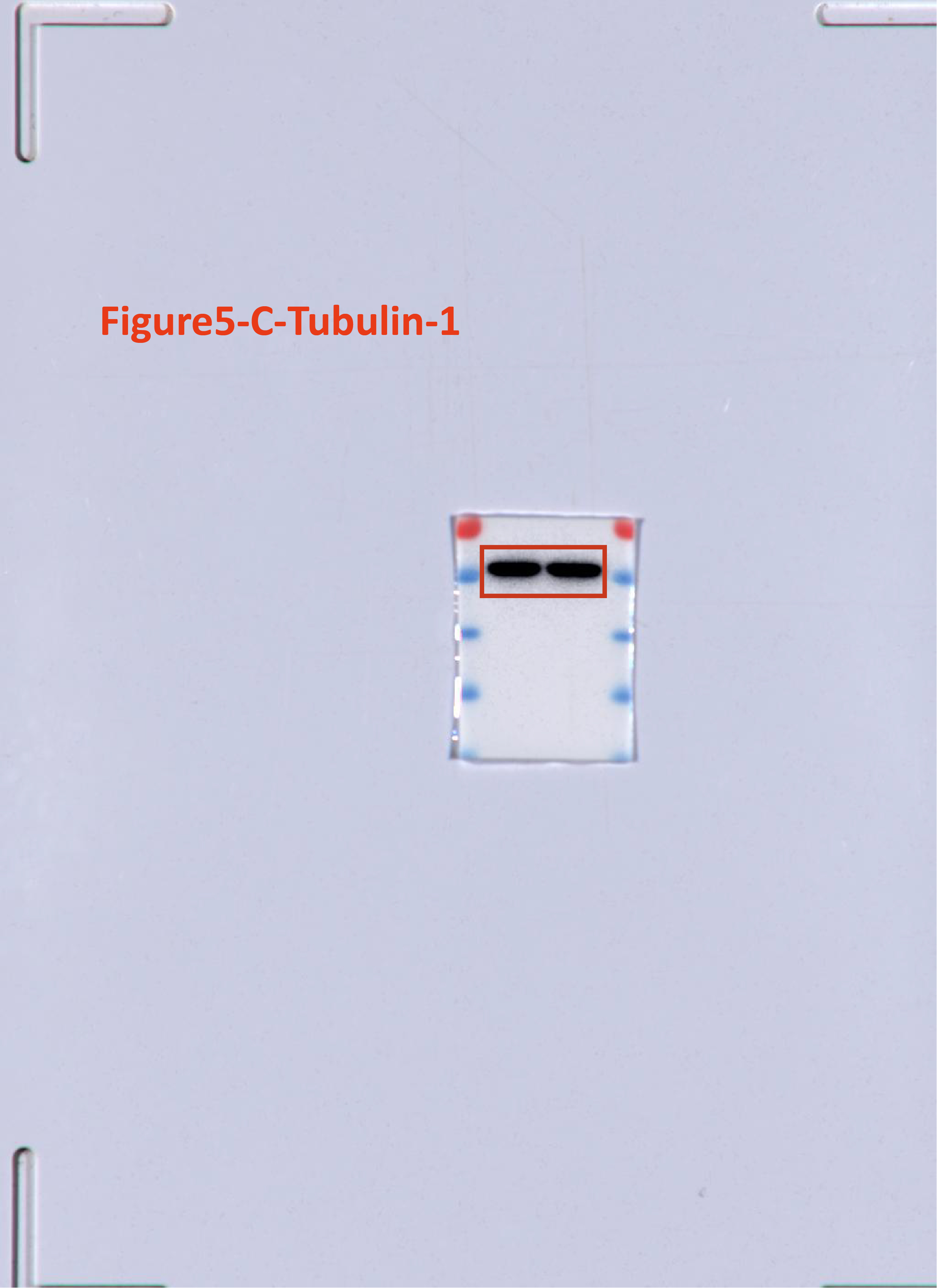

Supplement: Supplementary file 13 — Supplementary Information 13. [file 41598_2023_43744_MOESM13_ESM.jpg]

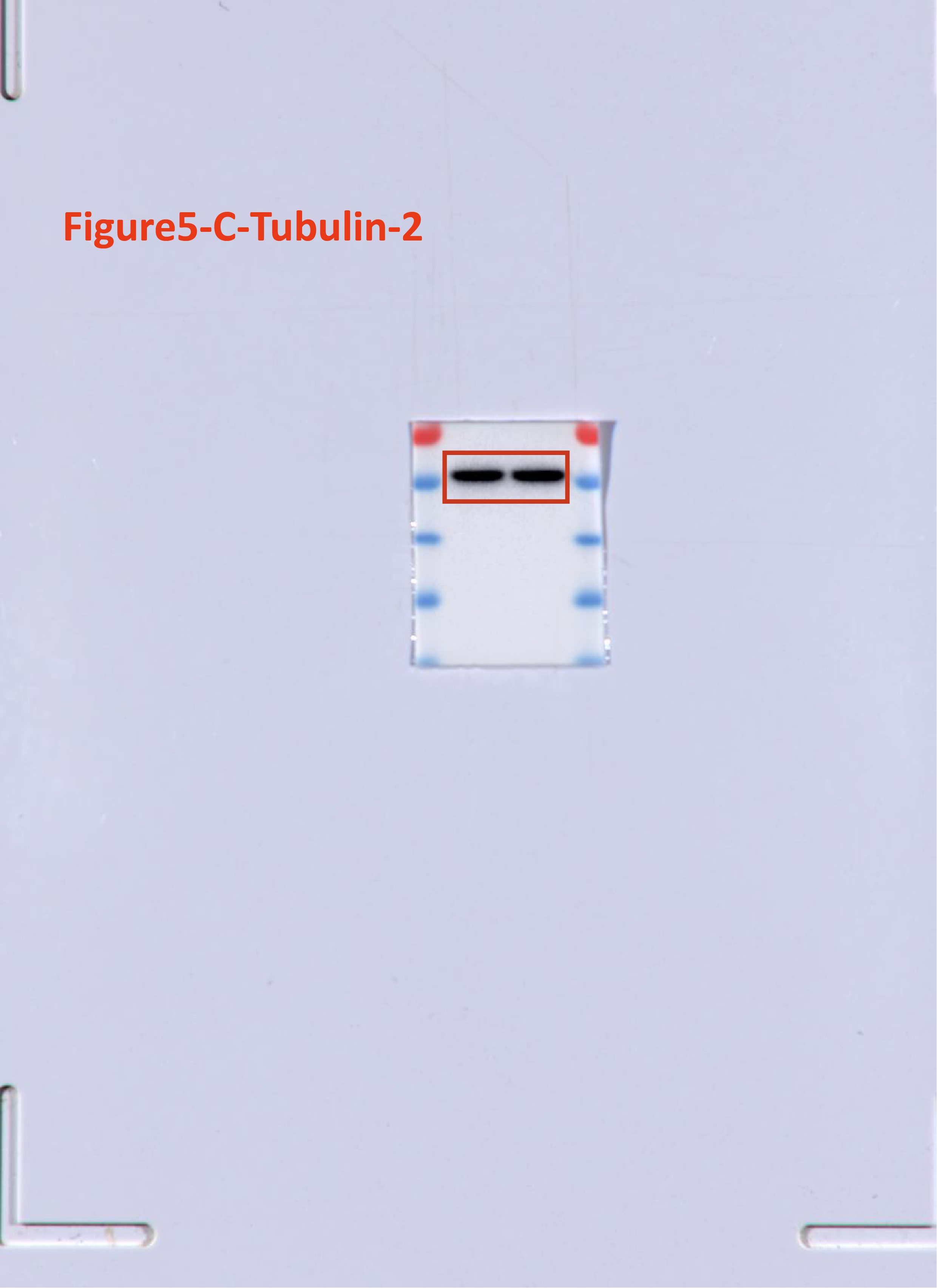

Supplement: Supplementary file 14 — Supplementary Information 14. [file 41598_2023_43744_MOESM14_ESM.jpg]

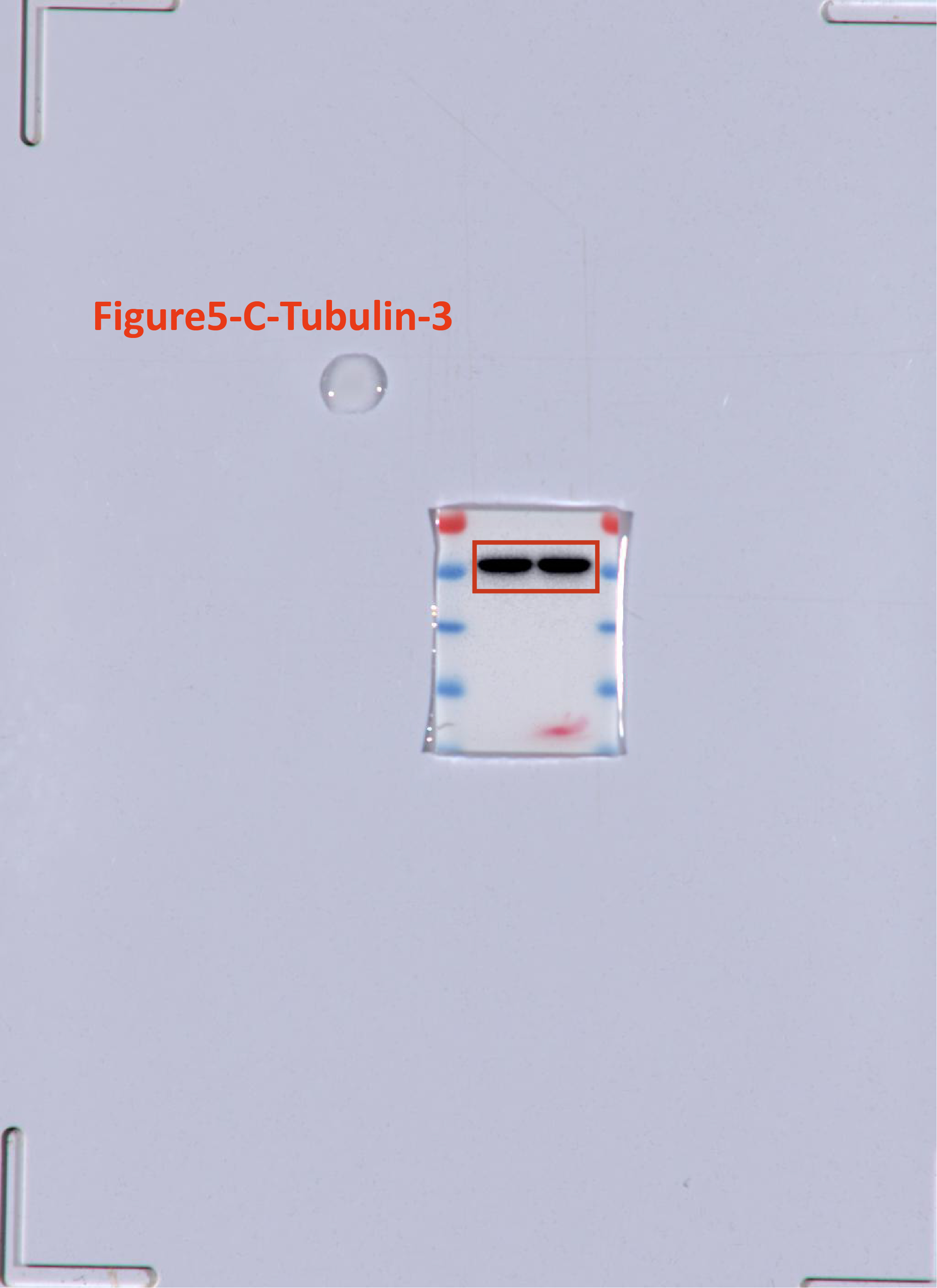

Supplement: Supplementary file 15 — Supplementary Information 15. [file 41598_2023_43744_MOESM15_ESM.jpg]

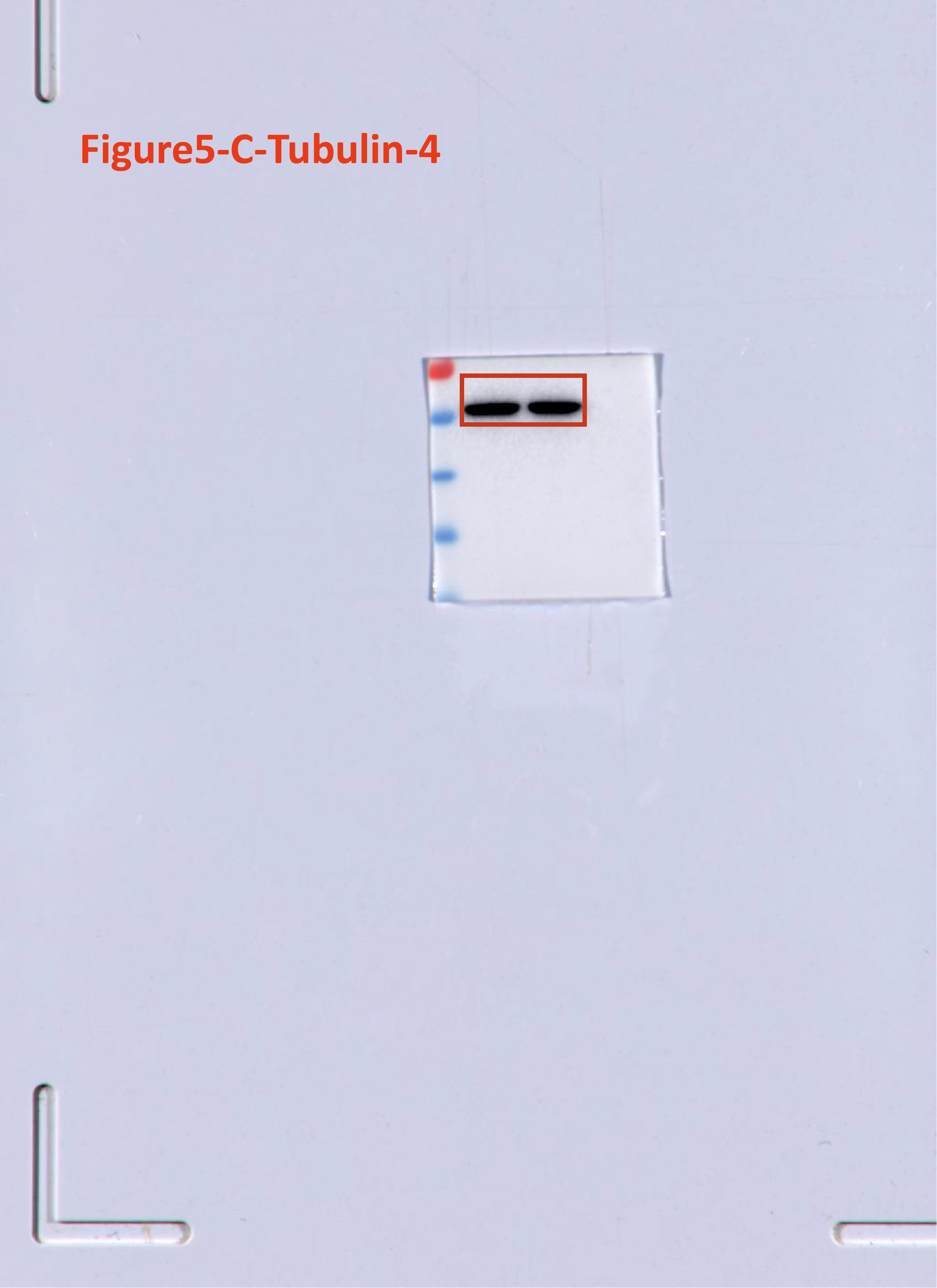

Supplement: Supplementary file 16 — Supplementary Information 16. [file 41598_2023_43744_MOESM16_ESM.jpg]

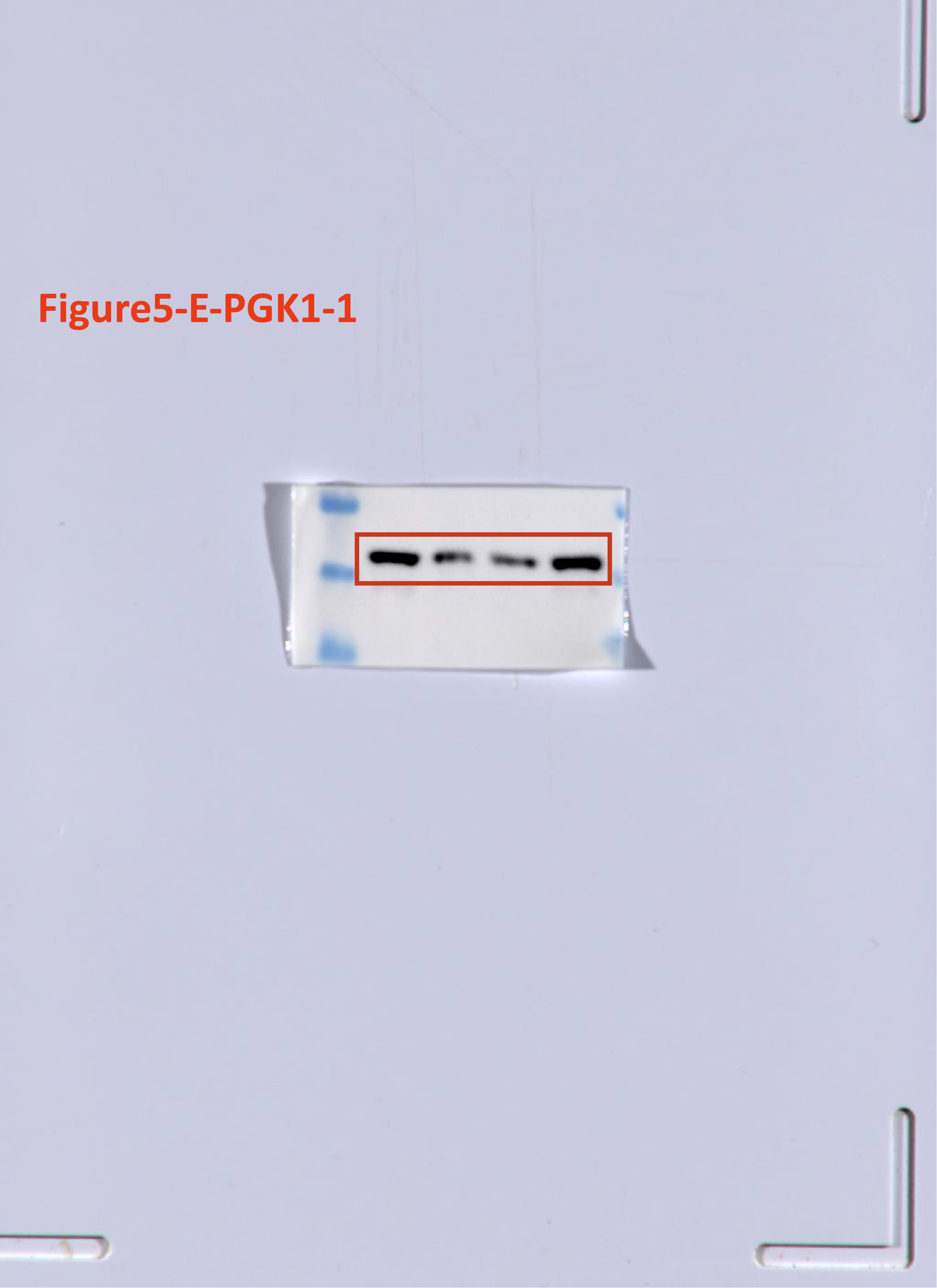

Supplement: Supplementary file 17 — Supplementary Information 17. [file 41598_2023_43744_MOESM17_ESM.jpg]

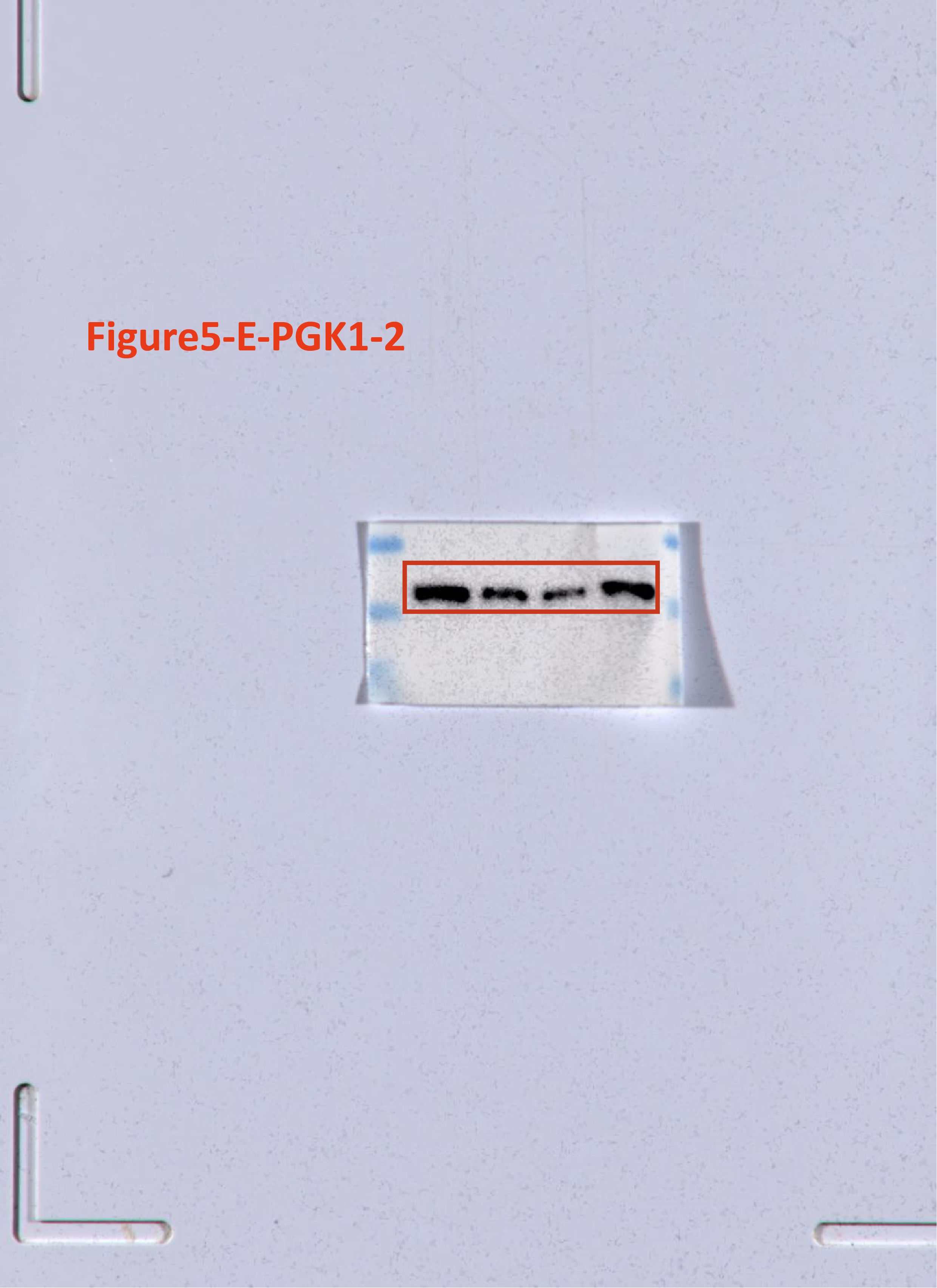

Supplement: Supplementary file 18 — Supplementary Information 18. [file 41598_2023_43744_MOESM18_ESM.jpg]

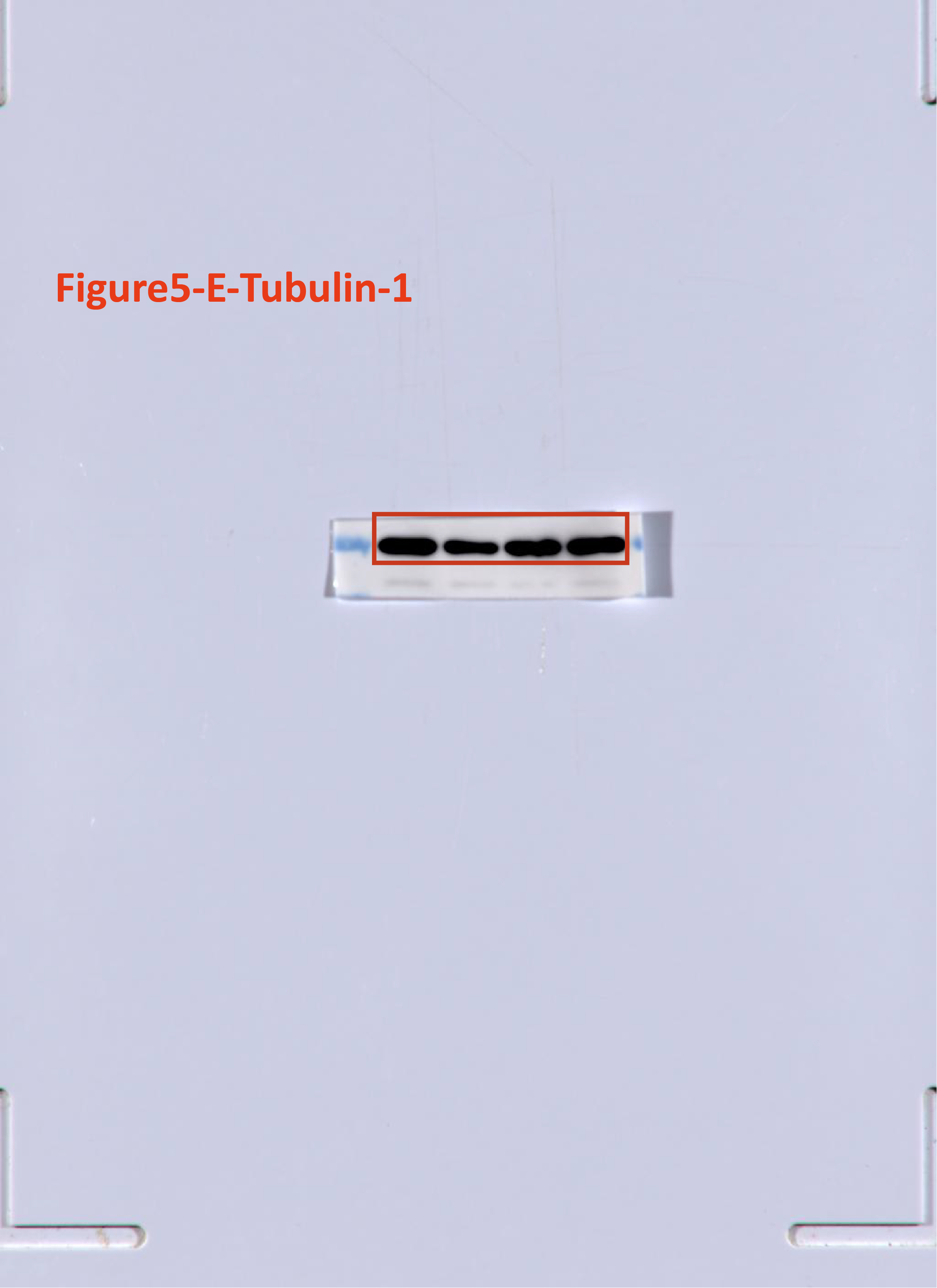

Supplement: Supplementary file 19 — Supplementary Information 19. [file 41598_2023_43744_MOESM19_ESM.jpg]

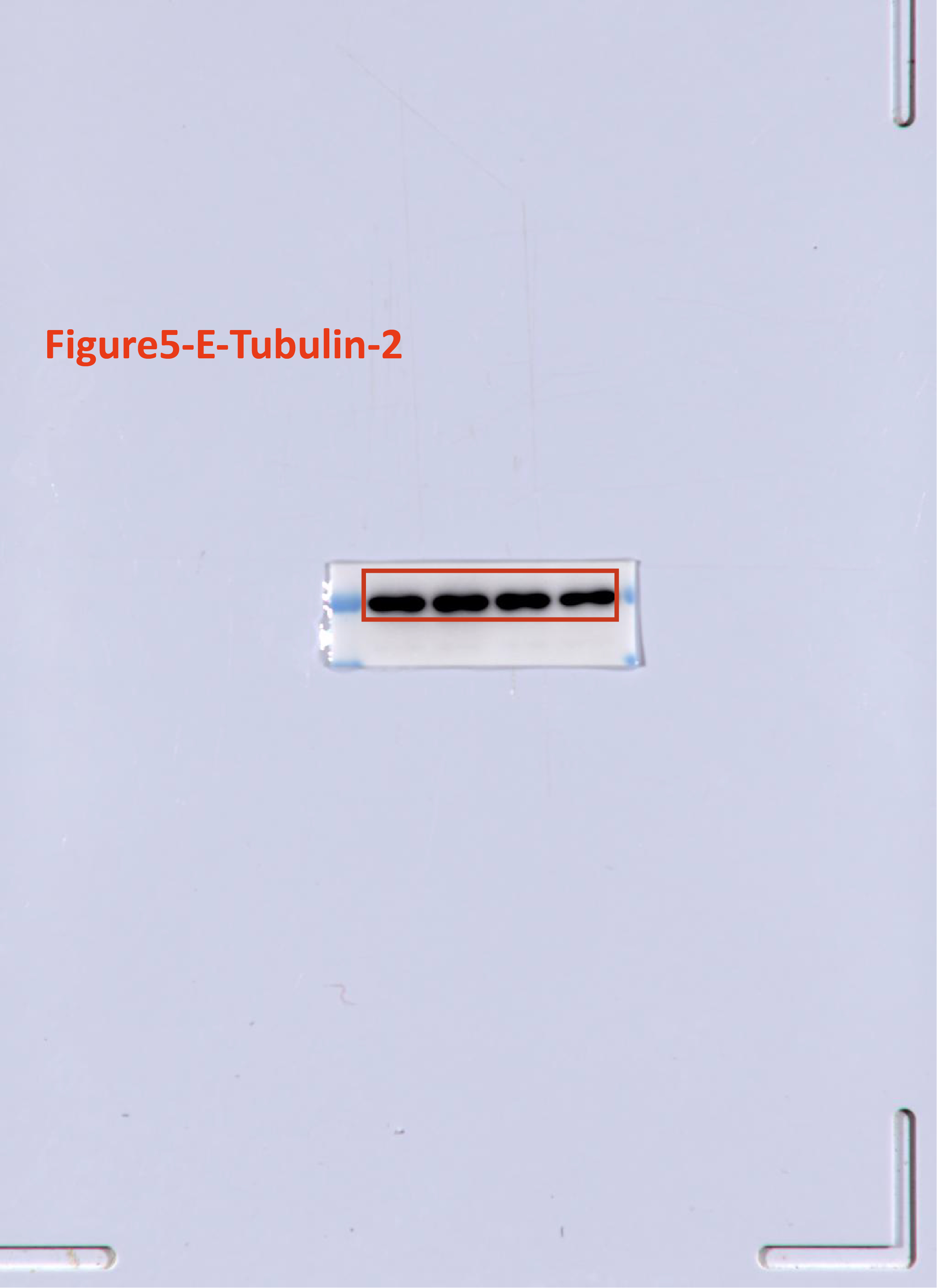

Supplement: Supplementary file 20 — Supplementary Information 20. [file 41598_2023_43744_MOESM20_ESM.jpg]
