# Supplementary material for: H19 encourages aerobic glycolysis and cell growth in gastric cancer cells through the axis of microRNA-19a-3p and phosphoglycerate kinase 1
Source: Sci Rep. 2023 Oct 11;13:17181. doi: 10.1038/s41598-023-43744-0 (PMC10567772; doi:10.1038/s41598-023-43744-0)
Supplement: Supplementary file 21 — Supplementary Information 21. [file 41598_2023_43744_MOESM21_ESM.docx]

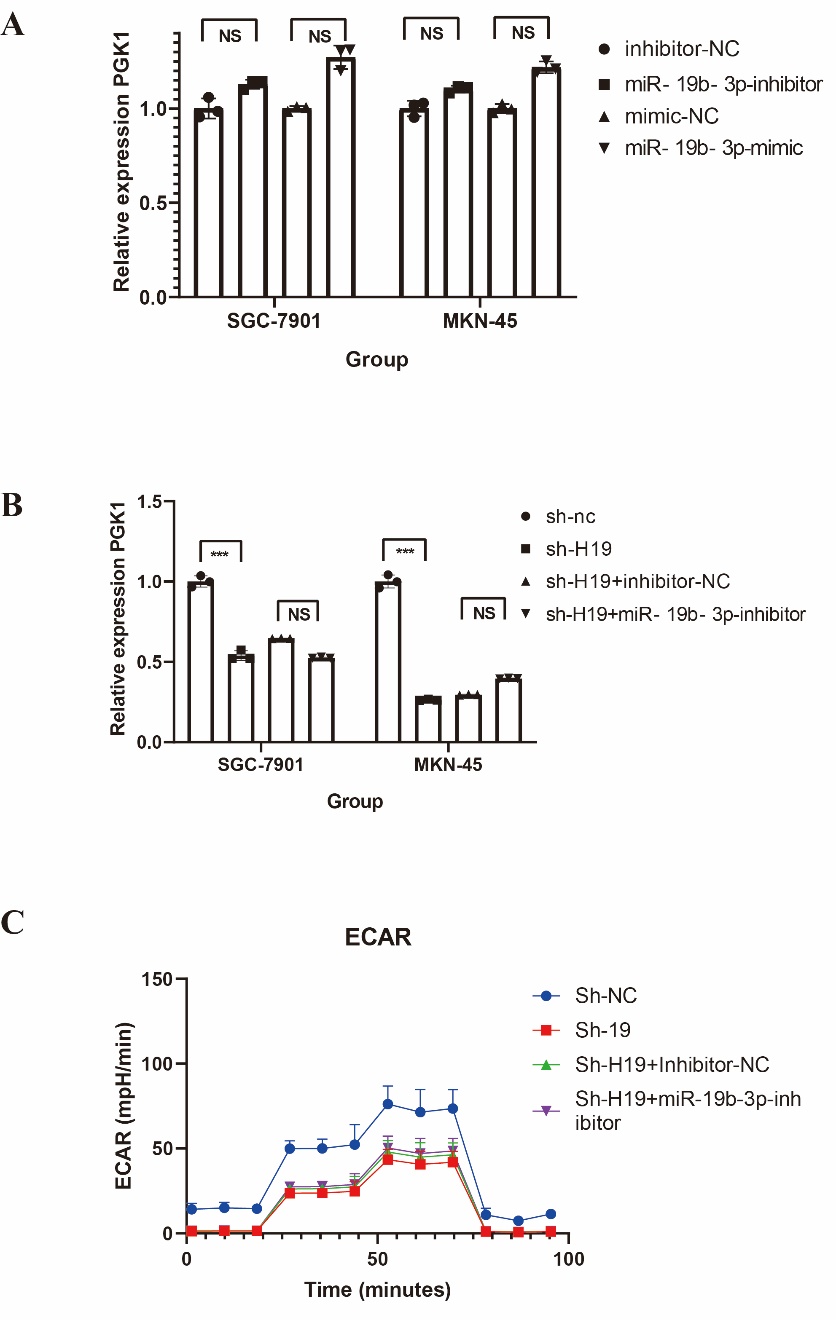


Supplementary Figure1

(A) Real-time quantitative PCR was used to assess the expression of PGK1 linked to glycolysis in SGC 7901 and MKN 45 cells after transfection with miR-19b-3p-inhibitor and miR-19b-3p-mimic. (B) mRNA levels of PGK1 were measured in both sh‐H19 SGC‐7901 and MKN‐45 cells after transfection with inhibitor NC or miR‐19b‐3p inhibitor. (C) ECAR assays showed on control, H19-knockdown, inhibitor NC and miR‐19a‐3p inhibitor in SGC-7901 cells. Each experiment was repeated at least three times with similar results. Data are presented as the mean ± SD and analyzed by Student’s t‐test. ns, not significant; *, P<0.05; **, P<0.01; ***, P<0.001.


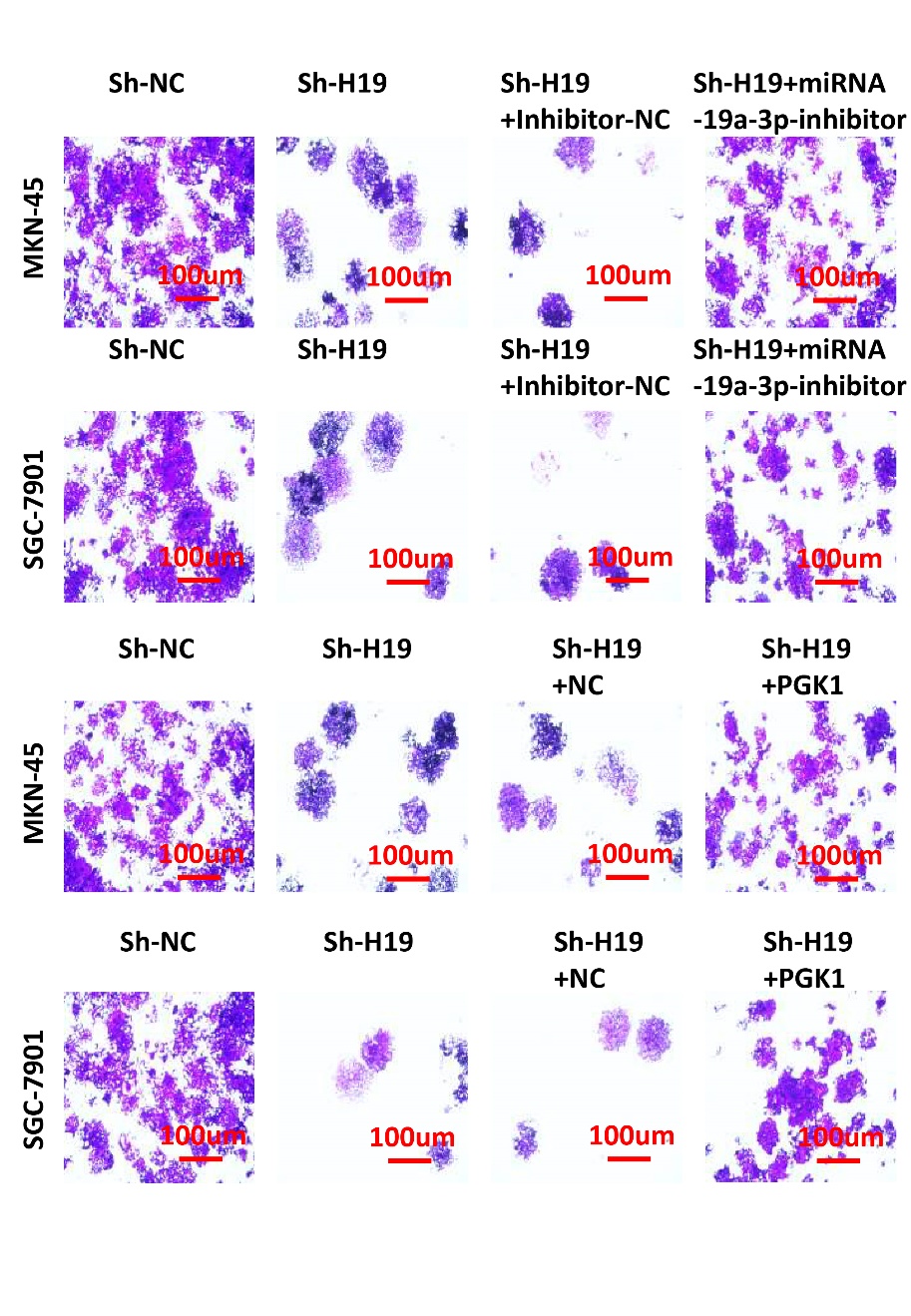


Supplementary Figure2

Colony formation assay of sh‐H19 SGC‐7901 and MKN‐45 cells after transfection with miR‐19a‐3p inhibitor or PGK1 overexpression plasmid. Scale bar is 100um.
